# Effect of Prenatal Docosahexaenoic Acid Supplementation on Blood Pressure in Children With Overweight Condition or Obesity: A Secondary Analysis of a Randomized Clinical Trial

**DOI:** 10.1001/jamanetworkopen.2019.0088

**Published:** 2019-02-22

**Authors:** Elizabeth H. Kerling, Jamie M. Hilton, Jocelynn M. Thodosoff, Jo Wick, John Colombo, Susan E. Carlson

**Affiliations:** 1 Department of Dietetics and Nutrition, University of Kansas Medical Center, Kansas City; 2 Department of Biostatistics, University of Kansas, Lawrence; 3 Department of Psychology, University of Kansas, Lawrence

## Abstract

**Importance:**

The blood pressure–lowering property of docosahexaenoic acid (DHA) in children and adults is known, and an observational study from the Netherlands has linked higher intrauterine DHA exposure to lower childhood blood pressure. However, the association of prenatal intake of DHA supplement with childhood blood pressure has not been evaluated in randomized clinical trials.

**Objective:**

To determine the effect of DHA supplementation during pregnancy on childhood blood pressure.

**Design, Setting, and Participants:**

This prespecified secondary analysis of the Kansas University DHA Outcome Study (KUDOS), a phase 3, double-blind, randomized, placebo-controlled clinical trial was conducted at several local hospitals in the Kansas City, Kansas, metropolitan area. Pregnant women (n = 350) were enrolled in the KUDOS trial between January 10, 2006, and November 17, 2009, and were followed up until their children were 18 months of age. During pregnancy, the women received either 3 capsules per day of placebo or 600 mg per day of DHA from a mean (SD) of 14.5 (3.7) weeks’ (all before 20 weeks) gestation until birth. The parents of 190 children consented to additional follow-up of their children until 6 years, which ended April 29, 2016. Study personnel involved in testing were blind to the randomization until all children had completed the trial. Data analysis was performed from May 23, 2017, to July 10, 2018.

**Interventions:**

Pregnant women were assigned to either 600 mg per day of DHA or a placebo that was half soy and half corn oil. Both placebo and DHA were provided in 3 capsules per day.

**Main Outcomes and Measures:**

Childhood blood pressure was a planned secondary outcome of a study powered to measure cognitive development. The hypothesis was that DHA would lower blood pressure prior to data analysis. Systolic blood pressure (SBP) and diastolic blood pressure (DBP) were measured at 4, 4.5, 5, 5.5, and 6 years and were analyzed for possible covariates using mixed models to generate a final model.

**Results:**

In total, 171 children (88 [51.5%] female) were included in this analysis. Of these children, 89 (52.0%) were randomized to the DHA group and 82 (47.9%) to the placebo group. A statistically significant interaction was found between treatment (placebo or DHA) and child weight status (5-year body mass index ≤85th or >85th percentile) for both SBP and DBP. Children who were overweight or obese whose mothers received placebo during pregnancy had higher SBP and DBP compared with children who were overweight or obese whose mothers received DHA (mean [SE] SBP, 104.28 [1.37] mm Hg vs 100.34 [1.02] mm Hg; DBP, 64.7 [1.23] mm Hg vs 59.76 [0.91] mm Hg). No differences in the SBP and DBP were found between children who were overweight or obese whose mothers received DHA and children who were not overweight or obese. In the mixed model analysis, the child’s age at blood pressure measurement and the maternal prepregnancy body mass index were the only other statistically significant variables (child age, SBP: *F* = 7.385; *P* = .001; DBP: *F* = 7.39; *P* = .001; prepregnancy BMI, SBP: *r* = 0.284; *P* = .001; DBP: *r* = 0.216; *P* = .01).

**Conclusions and Relevance:**

Maternal docosahexaenoic acid intake during pregnancy appeared to mitigate the association between childhood overweight condition or obesity and blood pressure.

**Trial Registration:**

ClinicalTrials.gov Identifier: NCT02487771

## Key Points

**Question:**

Is maternal intake of prenatal docosahexaenoic acid associated with reductions in childhood blood pressure?

**Findings:**

In this prespecified secondary analysis of a randomized clinical trial and follow-up of 171 mothers and their offspring, the children of women randomized to receive prenatal docosahexaenoic acid did not experience an increase in systolic blood pressure and diastolic blood pressure if they became overweight or obese compared with children of women randomized to placebo. A statistically significant interaction was found between prenatal treatment and child weight status.

**Meaning:**

Docosahexaenoic acid intake during pregnancy may protect children from the blood pressure–elevating consequence of childhood overweight condition or obesity.

## Teaser

This secondary analysis of a randomized clinical trial evaluates the risks and advantages of exposure to docosahexaenoic acid in utero for young children who are obese or overweight.

## Introduction

Consumption of docosahexaenoic acid (DHA) and eicosapentaenoic acid from fish oil is well known to reduce blood pressure (BP) in both adults and children.^1,2^ However, there has been recent interest in the potential programming association of DHA in utero and in early infancy with long-term physiological functions, including BP. Consistent with this possibility, a large population-based prospective cohort study from the Netherlands found an association between higher in utero DHA exposure and lower systolic BP (SBP) at age 6 years.^3,4^ Another 4-country European study found lower diastolic BP (DBP) at age 6 years in children randomized to an infant formula containing DHA for only the first 4 months of infancy.^5^

Only randomized clinical trials of DHA supplementation during pregnancy can determine if improving DHA nutrition during fetal life improves childhood BP. The Kansas University DHA Outcome Study (KUDOS) trial randomized pregnant women to either a DHA supplement of 600 mg per day or a placebo and followed up the growth and development of their offspring to 6 years of age. The follow-up included semiannual BP measurements from 4 to 6 years of age among 179 children (92 DHA and 87 placebo recipients) as an indicator of child health. Based on the findings of Forsyth et al,^5^ we hypothesized that maternal DHA supplementation would be associated with reductions in BP in childhood. Results from this randomly exposed cohort agree with those of the Forsyth et al^5^ report and 2 recent reports,^3,4^ suggesting that prenatal DHA exposure is associated with reductions in childhood BP; however, this report suggests that children who become overweight or obese are the ones protected from higher BP by maternal DHA supplementation.

## Methods

The University of Kansas Institutional Review Board approved this prespecified secondary analysis and both the parent randomized clinical trials, KUDOS (NCT00266825)^6^ and its follow-up (NCT02487771). The trial protocol is available as Supplement 1. Both the research protocol and written informed consent adhered to the Declaration of Helsinki.^7^ This study followed the Consolidated Standards of Reporting Trials (CONSORT) reporting guideline.

### Participants

From January 10, 2006, through November 17, 2009, 350 pregnant women were enrolled in the KUDOS trial, a phase 3, double-blind, randomized, placebo-controlled clinical trial of DHA supplementation delivered at several local hospitals in the Kansas City, Kansas, metropolitan area. Women were randomized to either placebo (3 capsules per day containing an equal mixture of soybean and corn oil) or DHA (3 capsules per day of algal oil that provided a total of 600 mg of DHA). Randomization schedules generated by the biostatistics department were for 2 maternal age groups (16-25.99 years and 26-35.99 years). Each 8–random number sequence included 4 assignments per group stratified by treatment. The Investigational Pharmacy at the University of Kansas Medical Center assigned women to placebo or DHA on the basis of age shared by study personnel. Both the placebo and DHA capsules were orange flavored and provided by DSM Nutritional Products. Women were enrolled at a mean (SD) of 14.5 (3.7) weeks’ gestation but had to be enrolled before 20 weeks’ gestation. The primary aims of the KUDOS trial were to evaluate the effect of prenatal DHA supplement of 600 mg per day, compared with placebo, on pregnancy outcomes and infant development to age 18 months.

**Figure 1.**
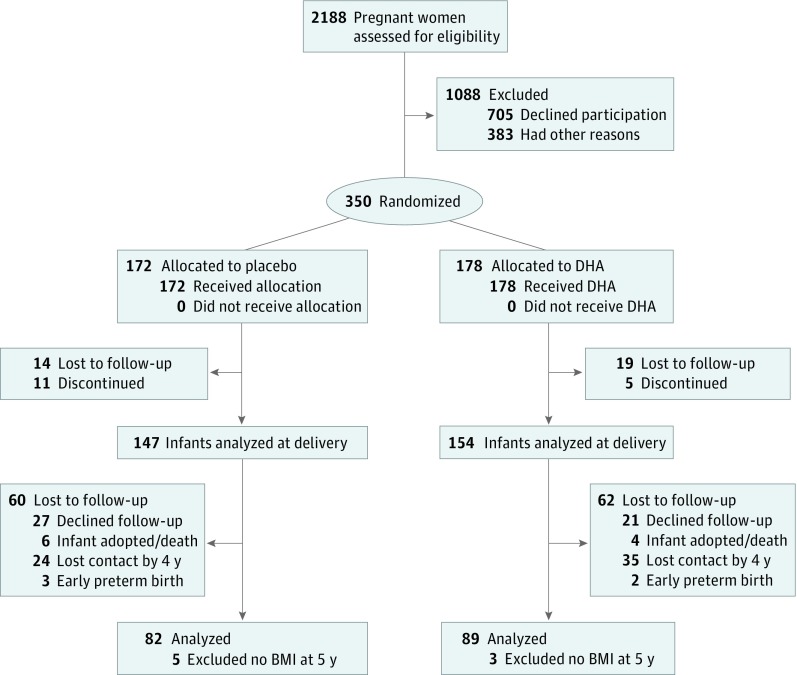
CONSORT Flow Diagram BMI indicates body mass index; DHA, docosahexaenoic acid.

When children were 18 months of age, the parents of 190 children gave permission for continued follow-up, which included longitudinal assessments of diet, cognitive development, BP, and growth to 6 years of age. Parents who consented to follow-up into childhood differed from those who consented to the KUDOS trial in that the mothers were approximately 1 year older at enrollment (26.2 vs 25.0 years), less likely to be black individuals (29.5% vs 41.5%), and more likely to be compliant with capsule intake (consuming a mean [SD] of 428 [117] vs 362 [150] capsules during the primary study).^6^ The follow-up study (conducted from July 17, 2008, to April 29, 2016) was powered on the primary aim of evaluating cognitive development. Dietary intake, growth, and BP were its secondary outcomes. Eleven of the 190 children (5.8%) were lost to follow-up before BP assessment began at 4 years of age. An additional 8 children did not have a body mass index assessment at 5 years of age. Thus, this secondary analysis included 171 children (Figure 1).

The principal investigator of the KUDOS trial (S.E.C.) was unblinded to report the results of pregnancy outcome after completion of the parent trial; however, all other study personnel remained blind to the randomization until after the results were entered for the last child who reached 6 years of age. Blood pressure was measured semiannually from 4 years through 6 years of age. Anthropometric assessment and 24-hour dietary recalls were obtained at each visit from birth to age 6 years. Inclusion and exclusion criteria of the KUDOS trial ensured that women did not have any health issue that could increase the risk for adverse effects on offspring growth and development. Exclusion criteria included preexisting diabetes, hypertension, HIV/AIDs, hepatitis, lupus, cancer, alcohol or other drug dependency, and BMI higher than 40 at the time of enrollment into the trial. Other details of study design and demographic characteristics were included in a report of pregnancy outcome.^6^ Information on childhood diet^8^ and 5-year body composition of the offspring^9^ has also been reported.

### Anthropometric Assessment

Height was measured without shoes to the nearest 0.1 cm using a wall-mounted stadiometer (Health O Meter; PORTROD). Body weight was obtained to the nearest 0.01 kg using the system integrated electronic scale. Anthropometric data were converted to BMI percentiles with Epi Info, version 3.5.4 (Centers for Disease Control and Prevention).

### Dietary Intake

A registered dietitian (E.H.K., J.M.T., or J.M.H.) trained in the multipass 24-hour dietary recall method and neutral probing questions interviewed parents about their child’s food intake at each visit throughout the KUDOS and follow-up trials. Multiple dietary recalls have been shown to represent usual dietary intakes.^10^ During infancy, the child’s dietary recalls were collected at 6 weeks and 4, 6, 9, and 12 months as well as from ages 1 to 6 years at 6-month intervals. Recalls were entered into the Nutrition Data System for Research, version 2008-2014 (University of Minnesota) and evaluated for accuracy by a second registered dietitian (one of E.H.K, J.M.T., or J.M.H.). If the parent or child was unable to recall 1 or more meals in a 24-hour period, the recall was excluded from analysis. The mean results of all 24-hour recalls obtained between 1 and 5 years of age were calculated to estimate the dietary sodium intake (grams per 1000 kcal).

### Blood Pressure Measurement

We measured childhood BP using an automated system (CARESCAPE V100 Monitor; GE), with the cuff size recommended for the child’s arm circumference. Blood pressure was measured in triplicate: the mean of the last 2 measures for SBP and DBP was taken unless the coefficient of variance was greater than 0.095. If the coefficient of variance was greater than 0.095 (9.2% of SBP and 19.4% of DBP measurements), we used the mean of the 2 closest measurements for each indicator. We excluded from analysis the results of assessments with only 1 reliable data point (n = 23). Irritated or restless children or mechanical failure were the main reasons for exclusion. Members of the staff who measured BP and made decisions related to data quality and calculations were blinded to the mothers’ study randomization.

### Statistical Analysis

Data analysis was performed (J.W. and J.C.) from May 23, 2017, to July 10, 2018. Children’s SBP and DBP measured at 4, 4.5, 5, 5.5, and 6 years of age were the dependent variables for this study. We conducted a type 3 test of fixed effects to test for the association of maternal randomization (placebo or DHA) and other independent variables with BP. Birth weight, mean sodium intake per 1000 kcal, days fed human milk, maternal gestational weight gain, and race and days smoked during pregnancy were eliminated as influential covariates. The secondary mixed model analysis included maternal DHA randomization, child weight status (BMI ≤85th or >85th percentile) at 5 years of age, child visit or age at assessment (4, 4.5, 5, 5.5, or 6 years), socioeconomic status, maternal prepregnancy BMI, and additional DHA supplements taken during pregnancy. The number of children studied and the number of valid BP assessments obtained at each age are shown in the eTable in Supplement 2. We chose weight status at 5 years of age because our analysis showed that children who were obese or overweight at age 5 years were, as a group, obese or overweight at 4 and 6 years of age. Two-sided *P* ≤ .05 for a 2-tailed analysis was considered statistically significant.

## Results

In total, 171 children (88 [51.5%] female) were included in this analysis. Of these children, 89 (52.0%) were randomized to the DHA group and 82 (47.9%) to the placebo group. The characteristics of children in the maternal randomization groups by weight status (BMI ≤85th or >85th percentile) are included in Table 1.

**Table 1.** Child Characteristics by Maternal Docosahexaenoic Acid (DHA) Randomization and 5-Year Weight Status^a^

Variable	No. (%)			
	Placebo Group at 5 y (n = 82)		DHA Group at 5 y (n = 89)	
	BMI >85th Percentile	BMI ≤85th Percentile	BMI >85th Percentile	BMI ≤85th Percentile
Sample size, No. (%)	20 (24)	62 (76)	32 (36)	57 (64)
Sodium intake, mean (SD), mg/d	2301 (609)	2016 (483)	2002 (470)	2028 (643)
BMI percentile, mean (SD)				
2 y	81 (28)	51 (27)	82 (15)	57 (23)
3 y	83 (16)	53 (24)	88 (12)	55 (25)
4 y	90 (9)	53 (24)	91 (7)	59 (26)
5 y	95 (4)	54 (22)	93 (4)	56 (22)
6 y	94 (5)	53 (23)	92 (7)	56 (24)
Human milk intake, mean (SD), d	137 (161)	206 (240)	202 (285)	213 (231)
Male, No. (%)	13 (65)	33 (53)	11 (34)	26 (46)
Female, No. (%)	7 (35)	29 (47)	21(66)	31(54)
Smoking during pregnancy, mean (SD), d	68 (102)	46 (96)	58 (108)	45 (94)
Prepregnancy BMI, mean (SD), kg/cm^2^	24.6 (4)	25.6 (5)	26.7 (5)	25.8 (5)
Birth weight, mean (SD), g	3381 (542)	3218 (511)	3447 (444)	3457 (515)

Abbreviations: BMI, body mass index (calculated as weight in kilograms divided by height in meters squared); DHA, docosahexaenoic acid.

^a^ No statistical comparisons were made.

Similar to those in the KUDOS trial cohort,^6^ children in the DHA group had a higher mean (SD) birth weight compared with the placebo group (birth weight: 3441 [485] g vs 3266 [522] g) (Table 1). Mothers of children who were overweight or obese in both treatment groups had a higher mean (SD) number of days of smoking during pregnancy (days smoking: 62 [106] d vs 45 [94] d). Mothers in the placebo group with children who were obese or overweight provided fewer mean (SD) number of days of human milk (BMI >85th percentile, 137 [161] days vs 202 [285] days) and their children consumed a higher mean (SD) amount of sodium (BMI >85th percentile, 2301 [609] mg/d vs 2002 [470] mg/d) compared with the DHA group that was obese or overweight (Table 1). However, it was apparent from examining the large variability in both human milk feeding and sodium intake that these differences were not statistically significant. In addition, our preliminary analysis eliminated these factors as important to childhood BP.

### Secondary Analyses

We tested a model for SBP and DBP that included maternal DHA randomization, child age and sex, and child BMI (≤85th or >85th percentile) at 5 years of age as well as important covariates (socioeconomic status, maternal BMI, and additional supplemental DHA taken during pregnancy). The aim of this analysis was to determine whether these additional factors mediated BP outcomes.

**Figure 2.**
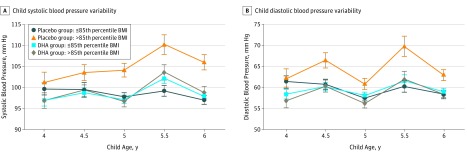
Systolic and Diastolic Blood Pressure at Each Age by Maternal Docosahexaenoic Acid (DHA) Randomization BMI indicates body mass index. Error bars indicate SE

Analyses of SBP yielded a marginal effect for DHA group and a substantial main effect for BMI (≤85th or >85th percentile). These associations were quantified by a statistically significant *P* for interaction = .04 (Table 2). Children who were obese or overweight (with a 5-year BMI >85th percentile) whose mothers were randomized to the placebo group had a mean (SE) SBP of 104.28 (1.37) mm Hg compared with obese or overweight children in the DHA group (100.34 [1.02]), a difference of 3.94 mm Hg. Maternal prepregnancy BMI was a statistically significant covariate with higher BMI associated with higher childhood BP (*r* = 0.284, *P* = .001). The analysis also yielded a statistically significant main effect for child age as SBP was higher at 5.5 years of age than any other age (*F* = 7.385; *P* = .001). For example, BP at 5.5 years in overweight condition and obese children in the placebo group was 110 (2.3) mm Hg compared with 4, 4.5, 5, and 6 years (101 [2.4], 103 [1.9], 104 [1.6], and 106 [1.8] mm Hg, respectively). The SBP and variability for each age by randomization and weight status are shown in Figure 2A.

**Table 2.** Systolic and Diastolic Blood Pressure as a Function of Maternal DHA Randomization and 5-Year Weight Status

Blood Pressure	Placebo Group (n = 82)		DHA Group (n = 89)	
	BMI >85th Percentile^a^	BMI ≤85th Percentile^b^	BMI >85th Percentile^b^	BMI ≤85th Percentile^b^
Sample size, No. (%)	20 (24)	62 (76)	32 (36)	57 (64)
Systolic, mm Hg, mean (SE)	104.28 (1.37)	99.43 (0.75)	100.34 (1.02)	99.30 (0.71)
Diastolic, mm Hg, mean (SE)	64.73 (1.23)	60.70 (0.67)	59.76 (0.91)	59.48 (0.64)

Abbreviations: BMI, body mass index (calculated as weight in kilograms divided by height in meters squared); DHA, docosahexaenoic acid.

^a^ These values represent the interaction between randomization group and weight status. The *P* for interactions = .04 for systolic blood pressure and *P* for interactions = .01 for diastolic blood pressure.

^b^ Individual value for SBP and DBP of the placebo group with BMI greater than the 85th percentile differs from the 3 other groups (*P* < .05).

Analysis of DBP yielded statistically significant main effects for the DHA group and BMI. These associations were quantified by a statistically significant interaction (Table 2), such that children who were obese or overweight had higher DBP only if their mothers did not receive the DHA supplement during pregnancy. Children who were obese or overweight (with a 5-year BMI >85th percentile) whose mothers were randomized to the placebo group had a mean (SE) DBP of 64.7 (1.23) mm Hg compared with children who were obese or overweight in the DHA group (59.76 [0.91]), a difference of 4.97 mm Hg. The analysis also yielded a statistically significant main effect for child age as DBP, like SBP, was higher at 5.5 years of age than at any other age. For example, mean (SE) DBP at 5.5 years in overweight condition and obese children in the placebo group was 69.8 (2.4) compared with 4, 4.5, 5 and 6 years (62 [2.3], 66.5 [1.8], 60.9 [2.3], and 63 [1.3], respectively). Higher maternal BMI appeared to be associated with higher DBP (DBP: *r* = 0.216; *P* = .01). The DBP and variability by randomization and weight status for each age are shown in Figure 2B.

## Discussion

The prevalence of high BP during childhood is on the rise, partly owing to the high rates of obesity during childhood. A study of more than 20 000 US children aged 2 to 17 years found 36% had high BP (>90th percentile for weight, age, and height) at least once in a year.^11^ A meta-analysis found that BP in childhood tracks to BP in adulthood^12^ and that higher BP in childhood is associated with risk for hypertension in adulthood.^13^ Some of us began measuring childhood BP in 2010 as part of monitoring the overall health of children born to women in the KUDOS trial and because a report of early postnatal DHA supplementation^5^ linked lower BP at age 6 years to higher DHA exposure. Subsequent reports from the Netherlands^3,4^ associating prenatal DHA exposure with lower childhood BP supported our hypothesis that children of women randomized to DHA supplementation would have lower BP.

We found a statistically significant difference in DBP, which was lower in the DHA group compared with the placebo group; however, childhood weight status emerged as a major factor in both SBP and DBP. In the mixed model analysis that included influential covariates, it became clear that the advantages from maternal DHA supplementation accrued only to children at risk for elevated BP owing to obesity or overweight condition. Both SBP and DBP showed a substantial interaction between maternal DHA randomization and child weight status, suggesting that maternal DHA supplementation exerted a protection for offspring who became overweight or obese in childhood. The increase in BP among children who were obese or overweight in the placebo group compared with the DHA group was large (3.94 mm Hg for SBP vs 4.97 mm Hg for DBP) and statistically significant. Although we knew that obesity or overweight condition is associated with higher BP in adults and children, we did not hypothesize a priori that children who were overweight or obese as a group would gain an advantage from intrauterine exposure to DHA.

We chose weight status at 5 years of age for the mixed model analysis as the midpoint in the ages of assessment. We realized that the weight status of individual children may have changed between 4 and 6 years of age, but the BMI of subgroups varied little among ages 4, 5, and 6 years as shown in Table 1. In fact, the groups who were obese or overweight at 5 years of age already had a higher BMI by 2 years of age than did their leaner counterparts from the same prenatal randomization. We doubt we would have observed an association between DHA supplementation and childhood BP had the incidence of overweight condition and obesity not been so high (29%) in this cohort of children. Nevertheless, the incidence is not markedly discrepant from the base rate in children in general in the United States, in which 1 in 5 school-aged children is obese.^14^

Forsyth et al^5^ did not report BMI or state that they adjusted for child BMI in their analysis. They reported 2.3 mm Hg lower SBP and 3.6 mm Hg lower DBP at 6 years of age in children randomized to a formula containing DHA for the first several months of life. If our findings are correct that the advantage of DHA supplementation for BP occurs only in children who are obese or overweight, we speculate that the Forsyth et al^5^ cohort may also have had a high proportion of children with overweight condition or obesity. Children in the Generation R study^3^ (n = 4455) were leaner than the children in our cohort: their mean BMI was just above the 50th percentile (actual BMI, 15.8; range, 13.6-21.2) at 6 years of age, whereas the mean BMI in this secondary analysis exceeded the 60th percentile at all ages. The authors of the Generation R study^3^ adjusted for BMI in linear regression models and still observed a substantial association between maternal ω-3 status and BP; however, the difference in BP was quite small.

Although smoking was variable within the subgroups, the mean number of smoking days during pregnancy appeared higher in mothers whose children were obese or overweight in both maternal randomizations. Maternal smoking appears to be associated with childhood obesity as has been shown previously.^15,16,17,18^

It is important to consider that the protection against BP in children who are obese or overweight during the preschool- and early school–aged periods is the result of a relatively brief intervention that occurred as much as 6 years earlier. A persistent physiological implication after an intrauterine intervention supports early developmental programming. Hypotheses derived from the Developmental Origins of Health and Disease model posit that less-than-optimal conditions or events experienced in utero are linked to adult disorders, including hypertension.

Furthermore, it is generally accepted that less-than-optimal in utero events do not necessarily result in adverse physiological outcome. Nordling^19^ first proposed a theory for cancer that Knudson^20^ termed a 2-hit or multiple-hit hypothesis. The hypothesis, then adopted by Li et al^21^ in relation to the Developmental Origins of Health and Disease model, is that in utero insults may only manifest as disease after a physiological second hit. Intrauterine malnutrition is a developmental insult. In the case of our findings, we suggest that less-than-optimal development of some organ system or systems associated with poor maternal DHA status is the first hit, which manifests as higher BP after the second hit of childhood overweight condition or obesity. We cannot know the biological mechanism, but evidence from 2 trials of DHA supplementation during pregnancy suggests 2 plausible mechanisms: intrauterine DHA resulted in more mature fetal autonomic nervous system^22^ and reduced cortisol response to a stressor in infancy.^23^ If these effects were prolonged after birth, either one could affect childhood BP.

Our results suggest an advantage of increased intrauterine DHA exposure to programming of BP response to excess body weight. The children studied in these trials were all fed human milk (a source of DHA) or infant formula with DHA (DHA was added to US formulas after 2002) exclusively or in combination. Consequently, we interpret these findings as an advantage of intrauterine DHA exposure for childhood BP. In the only other randomized clinical study that measured childhood BP after exposure to DHA early in development, Forsyth et al^5^ found that infants who were fed a formula with DHA for the first 4 months of life had lower SBP and DBP at age 6 years. Their results suggest that DHA exposure can program lower childhood BP for at least some period after birth.

The absolute SBP and DBP for the 4 randomization-weight status groups are shown in Figure 2. In searching the literature, we were unable to find chronological reports of BP in children at these ages. We believe these results may be of interest to those who will study BP in young children in the future.

### Limitations

A limitation of the study is that mothers in the placebo group with children who were overweight or obese provided a fewer number of days of human milk, and their children consumed more sodium than did children who were overweight or obese in the DHA group. Although these subgroup differences were clearly not statistically significant because of large SDs, they do suggest that subgroups of children may not be entirely comparable. Consequently, they limit the conclusion that DHA alone mitigated the association between overweight or obese weight status and childhood BP. The generalizability of this secondary analysis may be limited to children of mothers expected to have a healthy pregnancy and not be morbidly obese.

## Conclusions

Children of women randomized to DHA supplementation compared with those randomized to receive placebo during pregnancy appeared to be protected against the increase in SBP and DBP associated with childhood overweight condition and obesity.

## Notes

Supplement 1. **Trial Protocol**

Supplement 2. **eTable.** Number of Completed Visits and Number and Percent of Usable Blood Pressure Assessments by Group and Age

Supplement 3. **Data Sharing Statement**

## References

[1] Miller PE, Van Elswyk M, Alexander DD Long-chain ω-3 fatty acids eicosapentaenoic acid and docosahexaenoic acid and blood pressure: a meta-analysis of randomized controlled trials. Am J Hypertens. 2014;27(7):-. doi:10.1093/ajh/hpu024 24610882 PMC4054797

[2] Damsgaard CT, Schack-Nielsen L, Michaelsen KF, Fruekilde MB, Hels O, Lauritzen L Fish oil affects blood pressure and the plasma lipid profile in healthy Danish infants. J Nutr. 2006;136(1):94-99. doi:10.1093/jn/136.1.94 16365065

[3] VidakovicAJ, GishtiO, Steenweg-de GraaffJ, Higher maternal plasma n-3 PUFA and lower n-6 PUFA concentrations in pregnancy are associated with lower childhood systolic blood pressure. J Nutr. 2015;145(10):2362-2368. doi:10.3945/jn.115.210823 26246325

[4] Voortman T, Tielemans MJ, Stroobant W, Plasma fatty acid patterns during pregnancy and child’s growth, body composition, and cardiometabolic health: the Generation R Study. Clin Nutr. 2018;37(3):984-992. doi:10.1016/j.clnu.2017.04.006 28456538

[5] Forsyth JS, Willatts P, Agostoni C, Bissenden J, Casaer P, Boehm G Long chain polyunsaturated fatty acid supplementation in infant formula and blood pressure in later childhood: follow up of a randomised controlled trial. BMJ. 2003;326(7396):953. doi:10.1136/bmj.326.7396.953 12727766 PMC153849

[6] Carlson SE, Colombo J, Gajewski BJ, DHA supplementation and pregnancy outcomes. Am J Clin Nutr. 2013;97(4):808-815. doi:10.3945/ajcn.112.050021 23426033 PMC3607655

[7] World Medical Association World Medical Association Declaration of Helsinki: ethical principles for medical research involving human subjects. JAMA. 2013;310(20):2191-2194. doi:10.1001/jama.2013.281053 24141714

[8] Hidaka BH, Kerling EH, Thodosoff JM, Sullivan DK, Colombo J, Carlson SE Dietary patterns of early childhood and maternal socioeconomic status in a unique prospective sample from a randomized controlled trial of prenatal DHA supplementation. BMC Pediatr. 2016;16(1):191. doi:10.1186/s12887-016-0729-0 27884184 PMC5123236

[9] Hidaka BH, Thodosoff JM, Kerling EH, Hull HR, Colombo J, Carlson SE Intrauterine DHA exposure and child body composition at 5 y: exploratory analysis of a randomized controlled trial of prenatal DHA supplementation. Am J Clin Nutr. 2018;107(1):35-42. doi:10.1093/ajcn/nqx007 29381793 PMC5972598

[10] Johnson RM, Smiciklas-Wright H, Soucy IM, Rizzo JA Nutrient intake of nursing-home residents receiving pureed foods or a regular diet. J Am Geriatr Soc. 1995;43(4):344-348. doi:10.1111/j.1532-5415.1995.tb05805.x 7706621

[11] Beacher DR, Chang SZ, Rosen JS, Recognition of elevated blood pressure in an outpatient pediatric tertiary care setting. J Pediatr. 2015;166(5):1233-1239.e1. doi:10.1016/j.jpeds.2015.02.006 25919733

[12] Chen X, Wang Y Tracking of blood pressure from childhood to adulthood: a systematic review and meta-regression analysis. Circulation. 2008;117(25):3171-3180. doi:10.1161/CIRCULATIONAHA.107.730366 18559702 PMC3568631

[13] Chen X, Wang Y, Appel LJ, Mi J Impacts of measurement protocols on blood pressure tracking from childhood into adulthood: a metaregression analysis. Hypertension. 2008;51(3):642-649. doi:10.1161/HYPERTENSIONAHA.107.102145 18212267

[14] Hales CM, Carroll MD, Fryar CD, Ogden CL Prevalence of obesity among adults and youth: United States, 2015-2016. NCHS Data Brief. 2017;(288):1-8. 29155689

[15] Chen A, Pennell ML, Klebanoff MA, Rogan WJ, Longnecker MP Maternal smoking during pregnancy in relation to child overweight: follow-up to age 8 years. Int J Epidemiol. 2006;35(1):121-130. doi:10.1093/ije/dyi218 16260450

[16] Mendez MA, Torrent M, Ferrer C, Ribas-Fitó N, Sunyer J Maternal smoking very early in pregnancy is related to child overweight at age 5-7 y. Am J Clin Nutr. 2008;87(6):1906-1913. doi:10.1093/ajcn/87.6.1906 18541584

[17] Koshy G, Delpisheh A, Brabin BJ Dose response association of pregnancy cigarette smoke exposure, childhood stature, overweight and obesity. Eur J Public Health. 2011;21(3):286-291. doi:10.1093/eurpub/ckq173 21126981

[18] Currie LM, Tolley EA, Thodosoff JM, Long chain polyunsaturated fatty acid supplementation in infancy increases length- and weight-for-age but not BMI to 6 years when controlling for effects of maternal smoking. Prostaglandins Leukot Essent Fatty Acids. 2015;98:1-6. doi:10.1016/j.plefa.2015.04.001 25936840 PMC4444372

[19] Nordling CO Evidence regarding the multiple mutation theory of the cancer-inducing mechanism. Acta Genet Stat Med. 1955;5(2):93-104. 13301335 10.1159/000150766

[20] Knudson AG Jr Mutation and cancer: statistical study of retinoblastoma. Proc Natl Acad Sci U S A. 1971;68(4):820-823. doi:10.1073/pnas.68.4.820 5279523 PMC389051

[21] Li M, Reynolds CM, Segovia SA, Gray C, Vickers MH Developmental programming of nonalcoholic fatty liver disease: the effect of early life nutrition on susceptibility and disease severity in later life. Biomed Res Int. 2015;2015:437107. 26090409 10.1155/2015/437107 PMC4450221

[22] Gustafson KM, Carlson SE, Colombo J, Effects of docosahexaenoic acid supplementation during pregnancy on fetal heart rate and variability: a randomized clinical trial. Prostaglandins Leukot Essent Fatty Acids. 2013;88(5):331-338. doi:10.1016/j.plefa.2013.01.009 23433688 PMC3734850

[23] Keenan K, Hipwell A, McAloon R, Hoffmann A, Mohanty A, Magee K The effect of prenatal docosahexaenoic acid supplementation on infant outcomes in African American women living in low-income environments: A randomized, controlled trial. Psychoneuroendocrinology. 2016;71(9):170-175. doi:10.1016/j.psyneuen.2016.05.023 27290652 PMC4955755

