# Supplementary material for: Effect of Prenatal Docosahexaenoic Acid Supplementation on Blood Pressure in Children With Overweight Condition or Obesity: A Secondary Analysis of a Randomized Clinical Trial
Source: JAMA Netw Open. 2019 Feb 22;2(2):e190088. doi: 10.1001/jamanetworkopen.2019.0088 (PMC6484605; doi:10.1001/jamanetworkopen.2019.0088)
Supplement: Supplement 1. — Trial Protocol [file jamanetwopen-2-e190088-s001.pdf]

KUDOS FOLLOWUP, KUMC HSC #10146
(Revised Protocol – June 2010)

A. SPECIFIC AIMS

The purpose of this application is to extend an ongoing Phase III randomized clinical trial (RCT) funded under NIH 1 RO1 HD047315. The purposes of that trial is to determine whether increasing prenatal docosahexaenoic acid (DHA) intake will positively affect pregnancy outcomes, and whether such supplementation affects the development of visual acuity, visual attention, recognition memory, and various psychophysiological outcomes in infancy.

DHA is an essential long-chain polyunsaturated n-3 fatty acid that is present in every cell membrane in the body, but which contributes specifically to the structure and function of the CNS. In addition to being obtained postnatally from food sources, DHA is transferred prenatally from maternal stores to the fetal brain during the latter part of gestation. Given its role in the developing CNS, it has been broadly hypothesized that DHA has a role in sensory, cognitive, and intellectual development in infancy and childhood. While numerous RCTs have evaluated this hypothesis as a function of postnatal supplementation, studies of prenatal supplementation are particularly rare. As such, the existence of a cohort participating in an RCT on prenatal supplementation at a relatively high dose (600mg/day) of DHA represents a unique and important investment for the field of nutrition and development.

In the original application for this RCT, we proposed to follow a large sample of infants longitudinally until 18 months of age. As the third year of this RCT begins, we have consented 202 mothers, and project to recruiting through mid-2009 to attain our goal of consenting 350 mothers. However, because of the extended recruitment period necessary to accumulate this large sample, infants have begun graduating from the longitudinal schedule. The first infants reached the final assessment point of the original trial (18 months of age) in December of 2007. As a result, by the end of the funding period, there will be a sizeable number of preschoolers who will have participated in this RCT, but who will have been out of contact with the project for anywhere from 6 months to over 3 years. The scientific literature on longitudinal retention indicates that this condition will severely disadvantage maintenance of this sample for continuation of this study. Furthermore, the ages in which participants will be out of contact with the study and unmeasured (anywhere from 24 to 55 months) are particularly important ages in the area of development. These are the ages at which the higher-order cognitive functions emerge, and at which meaningful measures of basic intelligence can be validly assessed. Furthermore, at these ages, it is also possible to collect measures that directly bear on school readiness and various predictors of school problems. Therefore, the *overall aim of this application is to continue following this very important sample into the preschool period*, and begin to address the question of the long-term effect of prenatal DHA supplementation on very meaningful and adaptive measures of development.

The specific aims **of this application are**

1. To assess the effect of prenatal supplementation of DHA on measures that are characteristic of development in the early preschool period. These measures may be conceived as being grouped into four domains:

a. *Measures of higher-order cognitive function in preschoolers.* DHA is present in the frontal lobes (Neuringer et al.,1986) and may thus play a role in the development of higher-order cognitive functions. However, the effects of DHA supplementation on the later-emerging, higher-order cognitive abilities have never been assessed. We will assess the development of various aspects of higher-order functions that involve memory, rule learning, and inhibition in children 2 through 4 years of age who have been enrolled in this RCT.

b. Measures of language processing and preliteracy. During the ages covered by this supplement, it is possible to assess processes that are precursors to, and strong predictors of, reading and literacy. It has long been suggested that DHA may have meaningful outcomes that are related to school success and achievement; we can

address this question by administering both a comprehensive and a specific task in the fourth year of age for children enrolled in this RCT.

c. Adaptive Regulation. At these ages, it is also possible to address functional measures of adaptive regulation that are relevant to socioemotional development, attention, activity, and affect. These measures are relevant to outcomes in typically developing individuals, as well as in plotting the emergence of problem behaviors associated with ADHD and depression, both of which have been linked with DHA.

d. Global measures of intelligence. Despite the widespread belief that DHA may be related to improvements in fundamental cognitive outcomes, there have been relatively few studies using IQ as an outcome of DHA status, even fewer studies of DHA supplementation, and none of the effects of prenatal supplementation. At the ages covered by this supplement, we can implement an appropriate standardized tests at ages 3 and 4 to address this gap in the literature.

2. To assess health and diet in childhood. We will assess child anthropometrics, diet and blood pressure at each visit and record childhood illness through 6 years.

B. BACKGROUND AND SIGNIFICANCE

B.1. Docosahexaenoic Acid (DHA) and Development

Docosahexaenoic acid (DHA, 22:6n-3) is a long-chain polyunsaturated fatty acid (LCPUFA), a member of the n-3 fatty acid family. It is found in its highest concentrations in cell membranes, and because the accumulation of fatty acids in cell membranes is influenced by the kind and amount of n-3 acids in the diet, dietary fatty acids may influence all physiological functions.

Over the past decade, evidence has accumulated to suggest that DHA is important in during early development (Uauy et al., 2001). The central nervous system (CNS) is highly enriched in DHA and most DHA accumulation occurs during the last intrauterine trimester and in the first six postnatal months (Clandinin et al., 1980; Martinez, 1991). Given the putative importance of DHA to CNS integrity and function, it has long been suspected that DHA (and other LCPUFAs) play an important role in mammalian behavior and behavior. Omega-3 fatty acids affect learning and cognition in both animals (Carrie et al., 1999; Jensen et al., 1996; Wainwright et al., 1994) and humans (Isaev et al., 2000; Stevens et al., 1996; Willats, 2002; Willats & Forsyth, 2001; Willats et al., 1998a). Because DHA is accumulated in the brain and retina during gestation, it has been widely postulated that DHA may be have a major role in promoting early behavioral and cognitive function. The effects of DHA have been most intensively studied in early development, and during the early part of the life span, outcome variables have typically included measures of visual acuity, standardized test performance, and/or laboratory measures of early cognition.

B.1.1. Visual Acuity

Studies of infant visual function have been widely conducted in this area for some time (see reviews by Gibson & Makrides, 1999; San Giovanni et al., 2000a,b), showing both positive (Birch et al., 1992a,b,c; Birch et al., 1998; Carlson et al., 1996a; Carlson et al., 1996b; Carlson et al., 1993; Faldella et al., 1996; Makrides et al., 1995; Uauy et al., 1990) and null (Auestad et al., 1997; Bakker et al., 1999; Jensen et al., 1997; Innis et al., 1997) effects. Generally, when the effects of DHA are seen, they are typically manifest as an accelerated developmental function for visual acuity during the first year of life.

B.1.2. Standardized Tests

The results of studies using global assessments of cognitive function (e.g., the Bayley Scales of Infant Development, or childhood IQ tests, such as the Stanford Binet or Weschler tests) as outcomes of LC-PUFA supplementation or status are mixed between positive and null outcomes (Gibson et al., 2001). Some investigators have reported benefits on the Brunet-Lezine Scale (Agostini et al., 1995, 1997), the Bayley Scales (Birch et al., 2000; Carlson et al., 1994), and the Weschler Primary Preschool Scale of Intelligence (Birch et al.,

2007). On the other hand, null effects have been reported by Scott et al. (1998), Makrides et al. (2000) with the Bayley Scales and by Auestad et al (2003) with the Stanford Binet. A few studies of maternal DHA supplementation during pregnancy and/or lactation have been conducted, and although none of these trials (Helland et al., 2001; Jensen et al., 1998; Gibson et al., 1997) have reported advantages of DHA supplementation for infant development in the first year, the two that followed infants into the preschool period did report benefits (Helland et al., 2003; Jensen et al., 2004).

B.1.3. Specific Cognitive Tests

Several published trials show significant effects of DHA supplementation on the development of early information processing (Carlson & Werkman, 1996c; Werkman & Carlson, 1996). More mature infant/toddler attention has been reported in offspring of women who had higher RBC PL DHA at delivery (Colombo et al., 2004a; Willatts et al., 1998,b 2003). Innis et al. (2002) found that maternal milk DHA was associated with improved processing of speech contrasts (a critical skill in early receptive language development), a result that could be attributed to higher DHA accumulation in utero, given that higher milk DHA is evidence of generally higher maternal DHA status and an ability to transfer more DHA to the fetus relative to women with lower DHA status. Finally, a number of reports suggest that DHA status is related to improved performance on means-ends tasks at 10 months of age (Willatts et al., 1998a, Forsyth et al., 1998), which are generally thought to reflect the integrated functions several late-developing skills attributed to the maturation of the frontal cortex (Colombo & Cheatham, 2006).

B.2. The Case for Prenatal Supplementation

Relative to other cultural groups, US women have lower relative amounts of DHA in plasma, red blood cell phospholipids (RBC PL), and in breast milk than do women in cultural groups who consume higher amounts of DHA and EPA. For example, the average RBC PL DHA of US women in two recent studies was 4.91% (Smuts et al., 2003a) and 5.47% (Smuts et al., 2003b) DHA at the beginning of the 3rd trimester; this compares to a mean RBC DHA of 7.41% in Cuban women after parturition (Krasevec et al., 2002). It is estimated that the amount of DHA in breast milk in US women is 0.1 to 0.15% (Jensen et al., 1985); this compares to 0.45% for unsupplemented Norwegian women (Helland et al., 2003), and 0.43% for unsupplemented Cuban women (Kravesec et al., 2002). Indeed, US women have among the lowest breast milk DHA in the world. It has been widely assumed that these differences in DHA are attributable to differences in dietary intake (Otto et al., 1997); if this is the case, then DHA levels should be responsive to dietary supplementation. This position has enjoyed considerable empirical support over the last decade (Connor et al., 1996; Helland et al., 2001; Helland et al., 2003).

The transfer of DHA from mother to fetus appears to be enhanced during the last trimester of pregnancy as fetal and brain growth accelerates and DHA in the fetal brain accumulates. Perhaps as a result of this transfer, there is a small but consistent decline in average maternal RBC PL DHA seen during the last trimester (Smuts et al., 2003a, 2003b). Even in US women, where DHA levels are typically low, there is a wide variability in the range of maternal RBC PL DHA, and these individual levels tend to be remarkably stable across pregnancy (Smuts et al., 2003a, 2003b). Finally, these individual differences in DHA status within mothers during pregnancy appears to have longer-term effects on their infants, given that maternal DHA levels from pregnancy or at birth have been associated with individual differences in various measures of developmental outcome in their infants (Cheruku et al., 2002; Willatts et al., 2003; Colombo et al., 2004a). Individual differences in DHA status within mothers during pregnancy and lactation appear to have effects on their infants and children, given that maternal blood and milk DHA levels or infant DHA levels at birth have been associated with individual differences in various measures of developmental outcome in infants (Bakker et al., 2007; Cheruku et al., 2002; Colombo et al., 2004a; Innis et al., 2002, Innis and Friesen 2008; Jacobson et al., 2008; Malcolm et al., 2003; Willatts et al., 2003).

Finally, the available evidence suggests that DHA supplementation during pregnancy would, in fact, raise maternal levels by considerable amounts (Montgomery et al., 2003; Otto et al., 2000), making it probable that a prenatal supplementation would be effective.

B.3. The Current Randomized Clinical Trial (R01 HD047315)

Based on this thread of logic, in 2004 we proposed, and subsequently obtained NIH funding (R01 HD047315) for, the conduct of a double-blind randomized clinical trial (RCT) of maternal DHA supplementation with 600 mg DHA per day during pregnancy. Compliance with this dosage level was evaluated as part of the review process prior to the start of the RCT, and was found to be excellent.

The assessment schedules for the original RCT (and proposed as part of the original R01) are presented below. The first table shows contacts, measures, and evaluations conducted during the prenatal period. Several of the primary variables proposed to be studied reflected pregnancy health and outcome. For example, we hypothesized that supplementation would increase gestation, and that by increasing gestation length, we would see corresponding effects on birthweight and length. Our expectation of this chain of effects was based on prior data that had suggested that gestation length may be increased by prenatal supplementation with fish oil (Olsen et al., 1995, 2000; Olsen & Secher, 2002) or with a small increase in DHA (100 mg/d) from food (Smuts et al., 2003b); there are several plausible mechanisms through which such an effect might be realized. For example, DHA lowers blood pressure (Gerrard et al., 1991) and may thus reduce the probability of pre-eclampsia (D'Almeida et al., 1992; Fleischhauer et al., 1993; Williams et al., 1995). DHA may also increase gestation by counteracting processes leading to labor (Baguma-Nibasheka et al., 1999; Ma et al., 2000; Nair et al., 1997).

PRENATAL MEASURES	Baseline[b]	Monthly phone calls	Pre-term Hospitalizations	Delivery	Hospital discharge
Informed Consent	●				
Medical History	●				
Physical Examination	●			●	
Vital Signs	●			●	
Capsule consumption assessments		●		●	
Blood draw for DHA assessment (patient)	●			●	
Blood draw for DHA assessment (infant)				●	
Disease assessment	●		●	●	●
Pre-eclampsia / eclampsia assessment	●[a]			●	
Gestational diabetes assessment	●[a]		●	●	
Delivery (maternal) assessments				●	
Birth (infant) assessments				●	
Adverse events		●	●	●	●

[a] Exclusion criteria
[b]The baseline period is 8 to 14 weeks of gestation

A major focus of clinical trial, however, was to evaluate the effects of prenatal DHA on cognitive development in infancy. That is, we sought to study the infant/toddler/child development in infants born to women in the study by following them from birth through 18 months of age. The postnatal assessment schedule for the RCT is as follows:

POSTNATAL ASSESSMENTS	Postnatal Months					
	1.5	4	6	9	12	18
Visual evoked potentials	●	●		●	●	●
Visual habituation protocol with HR		●	●	●		
Attention span tasks					●	●
Distractibility protocol					●	●
Means-ends Problem solving task				●	●	
Bayley Scales of Infant Development II					●	●
MacArthur Communicative Development Inventory					●	●

The design of the postnatal protocols was based on the extant literature showing DHA as affecting electrophysiological measures of visual acuity (e.g., Birch et al., 1998), behavioral measures of visual habituation (Colombo et al., 2004), various psychophysiological and cardiac indices (Mozaffarian, 2005), higher-order problem solving (Willats et al, 1998a), standardized testing (Birch et al., 2000), and language (Innis et al., 1997). Measures were designed to be taken multiple times so as to provide a developmental function (Colombo, 2001b), and assessed at the point of maximal developmental sensitivity (Wainwright & Colombo, 2006). In addition, given that our laboratory was also engaged in a similarly-powered RCT on postnatal supplementation (e.g., Colombo et al., 2008), we sought to harmonize measures to ultimately allow some comparison of prenatal versus postnatal effects.

B.3.1. Status of the Current Sample

Our original plan was to consent 350 mothers into the supplementation study, with the aim of following a sample of 200 infants through to 18 months. We have currently consented 202 mothers into the prenatal supplementation. From the consented mothers, we originally projected that 60% would continue into the followup, and we are currently experiencing a success rate of 64%. To this point, 102 of the infants who have been delivered (note: some of the 202 mothers have not yet delivered) were enrolled in the follow-up study. Once infants reach the point of the follow-up study, we are experiencing very little loss (i.e., *our retention rate is 96%*). We currently have a follow-up sample in hand of 98 infants. The breakdown of those infants and their progress in the longitudinal schedule as of June 2008 is shown in the following table.

POSTNATAL ASSESSMENTS	Number of infants completed at each age
6 weeks	98
4 months	85
6 months	72
9 months	54
10 months	50
12 months	37
18 months	12

B.4. Rationales for this Application

Although the RCT as proposed has been impeccably conducted and is progressing smoothly, we are currently faced with a situation in which individual infants from this very valuable cohort may be lost unless we continue to keep them engaged in the study. Indeed, as the chart above shows, 12 infants have already graduated from the testing schedule, and the first few enrollees are in fact on track to turn 24 months this summer. This is occurring because the schedule of enrollment needed to accumulate the desired sample size will (as planned for in the original proposal) necessitate at least three years. As a result of this, we are still recruiting new subjects as the first infants recruited are aging out of the project and graduating from the longitudinal schedule.

We are on track to complete the projected testing to 18 months within the funded project period for R01 HD047315, but there are two problems. The first and most important aspect is that, as these toddlers approach the preschool period, they enter a time during which a number of extraordinarily interesting aspects of development will be emerging; these are aspects for which there is very good reason to believe that DHA has a role. This is an important period of development in a number of realms, and thus provides a unique opportunity to collect critical and meaningful data on the long-term impact of prenatal nutrition on childhood outcomes. A second issue is that it will be particularly difficult to continue following this sample into the elementary school years if we do not maintain and engage the cohort in some meaningful and regular manner. By maintaining a regular testing schedule (which, as we argue below, represents a "best practice" in the conduct of longitudinal research), we will keep the sample intact and allow for continuation of the project into the elementary school years, where we will be able to address questions of whether prenatal DHA supplementation affects school performance and overall behavioral adjustment.

In this supplemental application, we propose to do this by continuing to measure these infants on a semi-annual basis on a series of developmentally appropriate and scientifically meaningful measures from 24 through 48 months of age. The two rationales for this are described in detail below.

B.4.1. Scientific Rationale

The primary motivation for many studies of the effect of DHA on cognition is the belief that DHA supplementation may have enduring, long-term positive effects on cognition in childhood or beyond (Colombo, 2001b; Cheatham et al., 2008b). To this point, supplementation studies have been conducted in infancy, and the results of these have been generally promising (McCann & Ames, 2006), including those of specific abilities (and, in particular, those presented at 2008 meetings at ISSFAL in Kansas City; e.g., Colombo et al., 2008; Cheatham et al., 2008a). To this point, the infancy studies have served as surrogates or preliminaries for studies that address the fundamental underlying question of whether such supplementation affects variables that reflect actual adaptive, functional outcomes in childhood: higher-order cognition, mature intelligence (see Auestad et al., 2003; Birch et al., 2007), literacy and reading, school readiness, and adaptive function. The current cohort is about to move into those ages (i.e., late toddlerhood and early preschool) where it is actually possible to begin to address these questions by administering tasks and assessments that reflect these constructs directly. The extant literature on the effects of DHA on attention and other cognitive components has led many scientists to believe that DHA may also have a role in these higher-order abilities, and relate to school readiness, and functional abilities such as emotional/self-regulation and adaptive skills. A supplement to maintain this sample into the preschool years will allow us to test for these relations directly.

Part of the rationale for studying these abilities lies with the potential effects of DHA on the brain areas that mediate these higher-order skills and abilities. There is considerable evidence that suggest that DHA is present in the frontal areas of the brain (Salem et al., 2001) and is implicated in frontally-based behaviors (Greiner et al., 1999; Moriguchi et al., 2000). This strongly implies that this fatty acid has a role in mediating the meaningful higher-order cognitive, regulatory, and motivational constructs that emerge during the preschool period, and contributes to outcomes that are relevant to school and global functioning in childhood.

We consider four distinct areas of development that can be plausibly linked to DHA that are unambiguously accessible during the third and fourth years of life, which we propose to study through this supplement to the original project.

B.4.1.1. Higher-Order Cognitive Abilities ("Executive Function"). Specific fundamental cognitive functions emerge predictably at different points during the first year, most likely as a manifestation of maturation of particular brain areas (e.g., Colombo, 2001). For example, a young infant may be able to attend, to hold certain information in working storage, and to execute certain motor responses. However, as the infant *enters the second year and continuing through the preschool period*, these fundamental functions (e.g., attention, memory, motor response systems) (Colombo & Cheatham, 2006). This integration and coordination allows the toddler to be

capable of far more complex cognitive feats than those of the infant. For example, means-ends behavior (Moore & Meltzoff, 1999; Willatts, 1999) is the simplest of these; here, the child has the ability to hold a goal in working memory while coordinating attention and an appropriate motor response in order to attain a goal, or solve a problem. Other examples of this integrated cognition showing the emergent ability to use perceptually-based information about stimulus categories and integrate them with semantic networks that contribute to language (Booth & Waxman, 2002; Gopnik & Meltzoff, 1984; Waxman & Braun, 2005) also serve to support this point. Many of the tasks and abilities that are driven by this integrated cognitive function are characterized as "executive function," and it is widely believed that these abilities contribute to adaptive functioning (Roberts & Pennington, 1996) and are relevant to behavior problems, such as ADD/ADHD (Barkley, 1997, 2001).

It is widely believed that these integrative abilities are mediated by the development of the frontal lobes of the brain (Kane & Engle, 2002). The frontal lobes serve to foster cross-communication and coordination among areas subserving lower-order functions (Desimone & Duncan, 1995; Miller & Cohen, 2001; Thompson et al., 2005). The frontal areas of the brain are among the latest to develop, and the time course of their maturation coincides exactly with the emergence of these behaviors (Bell & Wolfe, 2007).

B.4.1.2. Emergent Literacy/Language Processing. Another critical area of interest involves the development of skills that represent the precursors of literacy. Generally, these precursors involve the basic aspects of language processing, and the primary and fundamental skills that are essential to the development of reading. These skills represent the construct of preliteracy, which is considered to be one of the major indicators of "school readiness" for preschoolers the US.

There is already evidence from the literature in infancy for the effects of DHA on language processing. Innis et al. (2001) studied 83 infants who were exclusively breast-fed for at least 3 months. Among the measures they collected was infants' performance on discrimination of speech sounds that are not present in the infants' native environments. The discrimination of such non-native speech is typically lost between 6 and 8 months of age, as a function of reduced plasticity in the language processing system. However, higher DHA levels in both erythrocite DHA and plasma phospholipid DHA levels measured at 2 months of age were positively related to infants' ability to discriminate these nonnative retroflex and phonetic speech tokens at 9 months of age.

In a more recent study, Lindmark and Clough (2007) investigated the effects of a DHA-rich fish-oil supplement on various tasks in a group of 20 children who had been formally diagnosed as dyslexic. The children were assessed at study intake and after 6, 12, and 20 weeks of supplementation. Although this particular study was not well-controlled, of special interest here was the use of measures that are diagnostic of reading and language disabilities, and the fact that there appear to have been significant gains on those measures across a time period during which one would not typically expect spontaneous or spurious improvement. Remarkable gains were observed for word decoding (speed of reading: 60%) and letter decoding (motoric-perceptual speed: 23%), and parents reported that the gains appeared to extend to generalized school achievement. The mechanisms through which DHA might lead to improvements in reading performance are unclear; individuals who have difficulty in reading typically share deficits in the decoding of the phonological aspects of language, as well as deficits in decoding visual input that is necessary for fluency in reading. Thus, difficulties in emergent literacy might be mediated by either simple visual mechanisms or more complex central ones. In one study of dyslexics supplemented with a DHA-rich fish oil, Stordy (2000) reported evidence for both mechanisms. Dyslexics supplemented for 1 month showed significant improvements in dark adaptation; after 4 months of supplementation, evidence for amelioration of the centrally-mediated motor difficulties (see Casebolt et al., 2002) that accompany dyslexia was also seen.

B.4.1.3. Adaptive Self-Regulation. Arousal and activation are generally considered to be behavioral components that are mediated by lower-order brain systems, such as the brainstem ascending pathways (e.g., Robbins & Everitt, 1995). There has been a considerable history linking the omega-3 fatty acids, and particularly DHA, to various neuropsychiatric disorders (see Young & Conquer, 2005, for a review), most notably disorders

involving mood and affect (Kidd, 2007; Stahl et al., 2008), and more recently disorders of attention (Richardson, 2006).

Within this context, DHA supplementation was initially considered as an intervention for ameliorating the symptomatology of these two types of disorders. Consistent with much of the extant literature on DHA as an intervention, the findings are mixed between beneficial and null effects for both depression (Stahl et al., 2008) and attention deficit disorder (ADD) or attention-deficit hyperactivity disorder (ADHD) (Hirayama et al., 2004; Sorgi et al., 2007; Stevens et al., 1996; Voigt et al., 2001; for reviews, see Richardson, 2006; Richardson & Puri, 2000). However, a consideration of the core functions leading to the symptomatology of these disorders provides a broader perspective on the the putative role of LC-PUFAs and DHA in the regulation of behavior. For example, the disorders of ADD/ADHD are largely considered to be attributable to deficits in self-regulation (see Barkley, 1997, 2000), which is itself another general function associated with the development of frontal lobe function. In this vein, it is possible to reconceptualize the role of DHA as one that serves to regulate emotion and behavioral activation, which would plausibly account for its relation to both disorders of mood and inhibition. This framework has been posited by Hibbeln et al. (2006). These authors suggest that early nutritional deficiencies in LC-PUFAs may alter set-points for various neurotransmitter systems, which in turn results in a cascade of neural events that ultimately results in impaired regulatory function by frontal lobe systems. Their model also features lower-order dysregulation of sympathetic responses to stress, which can be manifest as, for example, decreased heart rate (HR) variability. A similar model, in which early DHA deficiencies produce fundamental changes in neurotransmitter and brain structure that give rise to a broad matrix of symptoms in the domains of anxiety, aggression, and depression, is proposed by McNamara & Carlson (2006).

If even part of these models are accurate, we should see empirical associations between DHA and a broad variety of behavioral phenomena beyond disorders of attention and mood. Indeed, these models predict that DHA would be implicated in many processes where self-regulation or dysregulation is involved, including aggression, emotional regulation, and response to stress. Indeed, even a quick perusal of the extant literature indicates that there is considerable evidence for all of these.

For example, in an evaluation of the effects of LC-PUFAs on ADHD, Richardson and Puri (2002) used the Conners rating scales (e.g., Goyette et al., 1978). Although group comparisons showed an advantage for active treatment over placebo for all components of the Conners rating scales, the differences reached significance for three specific but yet widely discrepant subscales: inattention, anxiety/withdrawal, and disruptive behaviour. Thus, LC-PUFA supplementation reduced distractibility and improved focus, reduced disruptive behavior (i.e., increased regulation of behavior), but also improved participants' regulation of socioemotional stress. Hamazaki and colleagues provides a series of studies showing DHA as effective across the domains of aggression and responses to stress. For example, Hamazaki et al. (1996, 2004) show DHA supplementation reducing aggression in varying groups of people, although this reduction is most evident under conditions where participants are observed in relatively stressful conditions (Hamazaki et al., 1998; 2002). Sawazaki et al. (1999) and Hamazaki et al. (1999) showed that DHA directly mediates the response to stress in both human and animal populations. Thus, DHA can be conceptualized as improving self-regulation within the context of social interchanges (i.e., reduced aggression), and in the regulation of stress.

Buydens-Branchey et al. (2008) observed relatively strong correlations between an increase in plasma DHA and lower anger scores in a group of individuals engaged in a substance-abuse rehabilitation program. Finally, Irribarren et al. (2004) observed results that link high dietary intake of DHA and consumption of fish rich in n-3 fatty acids with lower hostility in young adults.

B.4.1.4. Intelligence. The discussion of the possible effects of DHA on overall intellectual development have bee broadly discussed (Colombo, 2001; Cheatham et al., 2006). Measures of IQ reflect a mixture of indices that are thought to reflect underlying "native" cognitive ability and to also represent "developed" or "acquired" ability (i.e., the individual's ability to profit from learning opportunities). There have been two studies of the

effect of postnatal DHA supplementation in infancy on overall intelligence and its associated subscales. One conducted on IQ in the preschool period with low levels of DHA (Auestad et al., 2003) showed no effects; another conducted on IQ during early elementary with higher levels of DHA (Birch et al., 2007) ages showed positive effects. There have been no studies of the effects of prenatal supplementation on IQ; this would be the first.

B.4.2. Longitudinal Rationale

A second rationale for the continued testing of participants from this cohort comes from the desire to maintain the sample for continuation into the elementary and school-age years. As noted above, widespread interest in the effects of DHA on child cognitive and intellectual development has led to the notion that DHA supplementation might well affect school performance and achievement. The ultimate direct evaluation of whether that is true will necessitate testing at elementary school ages, and the existence of this cohort allows an exceptional opportunity for such a test. It might also be noted that basic intellectual abilities, as reflected by IQ, generally settles into a stable trait at ages 7-9, from which individual differences remain essentially constant through adulthood (see Blaga et al., 2008; Colombo et al., 2008a,b). As such, we recognize that the cohort from this RCT is extremely valuable, and we propose steps to retain it for these future tests. This supplement will allow us to maintain meaningful contact with the cohort and allow for testing into the elementary school years in the continuation of this project. If we lose this cohort, however, such a test becomes improbable.

If we stop testing at 18 months, the first members of the sample will not be seen for over 3 years if we attempt to re-engage the sample when the grant is continued (these children would be 5.5 years old at that point). There is a sizeable literature on the science of longitudinal testing and retention, and that literature suggests that such a gap in testing would bode poorly for such a continuation. Obviously, the efficient and successful retention of longitudinal participants in research protocols over extended periods obviates or reduces issues of missing data (Menard, 1991) or selective attrition (e.g., Capaldi & Patterson, 1987; Oskamp et al., 1978; Winefield et al., 1990). As noted above, the topic of retention of participants in longitudinal studies has generated a considerable research literature on its own (e.g., Black & Holden, 1995; Elias & Robbins, 1991; Farrington, 1991; Julien et al., 1992; Menard, 1991; Vander-Stoep, 1999). Among the strategies that have been shown to improve retention in longitudinal studies are the fostering of strong relationships with participants (Bootsmiller et al., 1998; Young & Dombrowski, 1989; Given et al., 1990), staff persistence in establishing and maintaining contact with participants (Hartsough et al., 1996; Hough et al., 1996), the development of efficient and multilayered strategies for tracking of individuals (Ellickson et al., 1988; Ribisl et al., 1996), and the development of individualized materials to explain and justify the study to participants (Capaldi & Patterson, 1987; Ribisl et al., 1996). All these steps have been used to some degree to maintain the current RCT. However, the extant literature on longitudinal retention is uniformly consistent in indicating that the maintenance of regular contacts with participants is the most important step in maintaining longitudinal cohorts (Bootsmiller et al., 1998; Young & Dombrowski, 1989; Given et al., 1990).

B.5. Summary

To this point, we have sought to justify this supplemental application by explicating the role of DHA in cognitive development and the plausibility of a prenatal supplement, reviewing the state of the current RCT, and providing a rationale for testing the extant sample beyond 18 months based on (a) the importance of obtaining measures of higher-order cognition and adaptive/functional measures, and (b) the importance of maintaining the coherence of the cohort for future longitudinal study. In the next section, we outline the credentials of the team to carry out the proposed assessments, and provide additional data on the effects of DHA from a more recent RCT on postnatal supplementation to bolster the evidence of the effectiveness of DHA on cognitive components.

C. PRELIMINARY STUDIES

C.1. Principal Investigators' Expertise in Longitudinal Research

Drs. Carlson and Colombo have well-developed research programs and a extensive experience with longitudinal studies. They have worked together since 1993 and have published papers on infant followups from one previous RCT (Colombo et al., 2004a; Kannass et al., 2008). At the current time, they collaborate on two RCTs on maternal-infant outcomes as a function of DHA supplementation at the KUMC laboratory. The NIH-funded RCT on prenatal supplementation on which this application is based is one of these; another is an industry-funded RCT on postnatal supplementation that is currently ongoing and is following a cohort out to age 7. In addition, Colombo is collaborating on a third RCT (NIH R01 HD41184) on the effects of zinc supplementation on early cognition in Lima, Peru with Drs. Laura Caulfield (Johns Hopkins University) and Nelly Zavaleta (Instituto Investigaciones Nutritional).

Prior to these collaborative efforts, both PIs have long independent histories of longitudinal research in their own rights. Carlson has studied the effects of LC-PUFAs on preterm and term infants and in pregnancy, and outcomes such as visual acuity, and some cognitive outcomes in young infants (e.g., Carlson et al., 1991, 1993, 1994, 1996a,b,c; Smuts et al., 2003a,b; Werkman & Carlson, 1996). Colombo has conducted longitudinal research on the psychometrics and predictive validity of various measures of infant cognition over the last 20 years (e.g., Blaga et al., 2008; Colombo et al., 1987a,b, 1988, 1989, 2002, Moss et al., 1989; Saxon et al., 1996, 1997), most recently a longitudinal study of over 200 infants measured intensively over the first year, and then out to preschool age to determine whether particular patterns of change in attention relate to preschool IQ and language performance (Colombo et al., 2004b, 2008a,b). That particular study was marked by a retention rate of nearly 80% over a 3 year period, through the implementation of various efforts to maintain the sample (infant t-shirts, newsletters, frequent mailings, provision of individualized reports, and open email channels for parents to request information and/or advice).

C.2. New Data on DHA effects on Cognitive Outcomes

Although the current prenatal-supplementation RCT is still blinded and no outcomes are available from the study yet, the other, industry-funded postnatal supplementation RCT was unblinded and findings from that study were recently presented at the 2008 ISSFAL meetings in Kansas City. In that RCT, we are attempting to determine whether dietary supplementation in infancy with varying levels of DHA is related to basic psychophysiological indices and heart-rate (HR) defined measures of visual attention across the first year of life. Here, Infants were randomly assigned to formulae containing 0% DHA, 0.33% DHA, 0.67% DHA, or 1% DHA from birth to 12 months of age. They have been tested through to 18 months in various tasks, but the available data are from the visual habituation protocol (a common assay for visual learning in human infants; see Colombo et al., 2004a) at 4, 6, and 9 months of age. HR was measured simultaneously with visual responses in the protocol (see Colombo et al., 2001), as this provides a means for discriminating different types of attention from simple visual responses (Colombo, 2002; Richards & Casey, 1992). Specifically, HR deceleration during looking (see accompanying figure above) is characterized as *sustained attention* (SA), and is thought to reflect the most active phases of infants' encoding and learning (Richards & Casey, 1992).

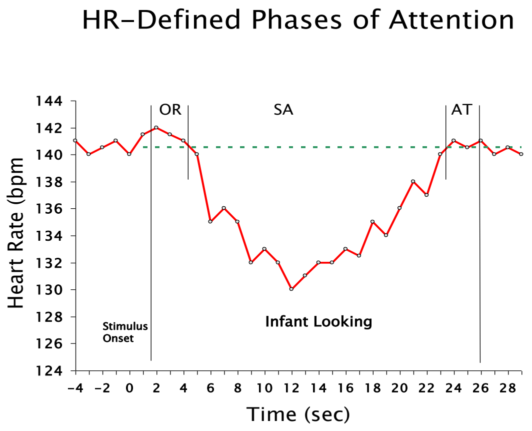

We were able to gather a relatively large longitudinal sample (*ns*=102, 103, and 84 respectively at 4, 6, and 9 months), and subject the data to mixed-model techniques that utilize all the available data from the longitudinal design. As expected, infants' HR declined across ages (all but one Age effect attained *p*<.001 levels of significance). Of some interest were the physiological effects of DHA; infants assigned to consume formula with *any* level of DHA showed consistently lower HR (by approximately 5 bpm) than infants assigned to the unsupplemented (i.e., 0% DHA condition). This effect emerged in the analysis at each age, and in the analysis of each phase of the habituation paradigm (see figure at left), with all Group effects attaining at least *p*<.002 levels of significance.

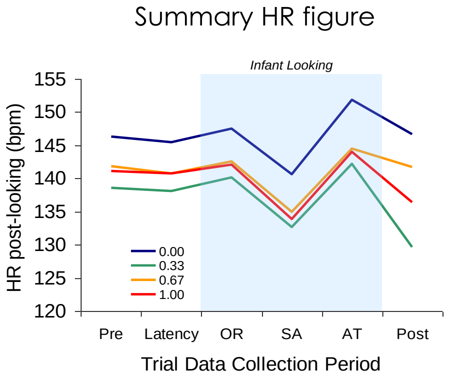

Of particular relevance to the consideration of later cognition was the fact that infants fed DHA-supplemented diets also showed higher proportions of their time in the phase of Sustained Attention than infants assigned to the 0.00% DHA condition (Group effect, *p*=.025); again, there was no dose-response, and the three groups receiving *any* DHA performed equivalently, and each was different from the unsupplemented (0.00%) condition. The means for sustained attention (collapsed across age) are shown in the accompanying figure at right. Thus, supplementation increased the proportion of sustained attention during the first year. This is significant, because recent analyses from our other longitudinal work (Colombo et al., 2004b, 2008b) suggests that a profile in which sustained attention remains high which implies efficient processing and has been associated with improved preschool cognitive and language performance. Thus, these data again show considerable promise for the efficacy of DHA, and for the production of enduring, long-term effects that we anticipate being able to demonstrate with the tasks administered with this supplemental application.

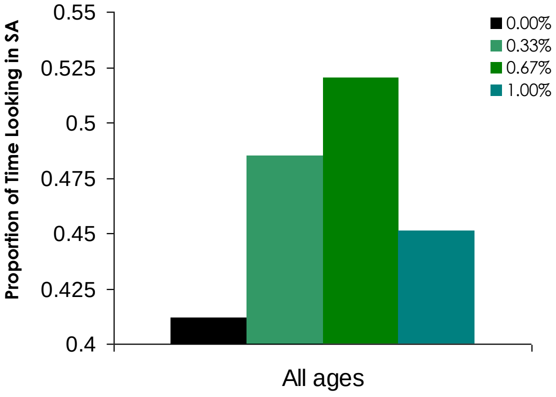

D. RESEARCH DESIGN AND METHODS

D.1. Design

The proposed supplemental measures are added to the randomized, double-blind, placebo-controlled Phase III Clinical Trial as described in R01 HD047315. In that RCT, enrolled patients are randomized to consume either capsules of an algal oil as a source of DHA or soybean oil (which does not contain DHA). Capsules are provided for consumption of two capsules daily across the second and third trimesters of pregnancy; the experimental group received 600 mg of DHA per day. The placebo group receives the same amount of soybean oil with 120 mg α-linolenic acid (a precursor of DHA) per day, and intake is evaluated monthly during the second and third trimester of pregnancy.

After enrollment, participants are contacted by telephone every four weeks until delivery and asked about their capsule consumption and spontaneous reports of adverse experiences were recorded. They are reminded at

that time of additional capsules being mailed to them, of the need to return any unused capsules from the previous month; these returned capsules are sent to the Investigational Pharmacy where they are counted, recorded, and destroyed. At delivery, study assessment data are collected for patients and their infants.

Enrollment has been taking place on a continuous basis. We anticipated that enrollment would require 3 years; our current analyses suggest that it will take approximately 3.5 years. We are on track to meet our projected total of enrolling 350 consented patients (175 per treatment group) in order to obtain 260-280 patients (~130-140 per group) with data that can be evaluated.

D.2. Study population

Women are being enrolled if they are 16 to 31 years of age and are in their 8th to 20th week of gestation (based upon dates or ultrasound). Inclusion and exclusion criteria are similar to a previous trial (Smuts et al., 2003a) and are illustrated in the table below.

Inclusion Criteria	
1	Pregnant females 16.0-35.0 years of age (inclusive) at 8-20 weeks gestation at enrollment (date/ultrasound)
2	Agree to consume study capsules from enrollment until delivery
3	Agree to return to the study center for delivery
4	BMI < 40
5	No serious illnesses (e.g., cancer, diabetes, lupus, hepatitis, sexually transmitted diseases, not HIV positive)
6	Available by telephone
Exclusion Criteria	
1	Less than 16 or greater than 35 years of age
2	BMI < 40
3	Serious illness such as cancer, lupus, hepatitis, sexually transmitted disease or HIV positive
4	Expecting multiple infants
5	Diabetes or gestational diabetes at baseline
6	Elevated blood pressure due to any cause
7	Not planning to return to the study center for delivery
8	Gestational age at baseline < 8 weeks or >20 weeks
9	Unable or unwilling to agree to consume capsules until delivery
10	Unable to provide informed consent in English

D.2.1. Sample Projections and Realizations

Projections. Of the estimated 350 evaluable patients we intend to consent at delivery and who remain eligible to continue in the followup, we had anticipated having about 200 mothers continue in the postnatal follow-up. Based on our past experience with this population (Colombo et al., 2004a; Colombo et al., 2004b), we had expected 50% attrition by the end of the postnatal follow-up, which we projected would allow us to finish the study with at least 100 children across the two study groups.

Realizations. Although recruitment is occurring somewhat slower than we had anticipated (we will likely need to recruit for 3.5 years, rather than 3.0 years), retention in the current trial has been excellent and has actually exceeded our projections. The target sample size for the trial was 350 women; we have experienced only approximately l6% dropout during the prenatal phase and thus estimate that 294 women will complete the prenatal phase and enroll their infants in the postnatal phase. However, we are currently experiencing less than

10% attrition through 18 months of the postnatal followup (i.e., we have lost 4 of 98 postnatal enrollees to this point). While the original projections were designed to allow for sufficient power for the postnatal follow-up studies through 18 months (see section D.7. below), the current attrition rate essentially guarantees that the extended followup will be powered appropriately. We now expect that at least 200 participants (i.e., at least 100 per group) will complete the current trial to 18 months. This is *double the expected sample size* that we originally projected, and this will provide excellent power for a continuation, as we estimate power of .95 to detect even a modest effect size.

D.3. Study Product, Randomization and Records of Product Use

D.3.1. Study product

The experimental group receives capsules containing a marine algal oil source of DHA (DHASCO, Martek Biosciences, Columbia, MD) that has been recognized as GRAS for US infant formulas and that is self-affirmed GRAS for food. The placebo group receives capsules containing soybean oil. Both capsules are prepared and provided without cost by Martek Biosciences, Columbia, MD. The capsules are provided in bottles of 60 capsules (a 30-day supply). The algal oil provides 600 mg DHA/2 capsules or 600 mg/day; soybean oil does not contain DHA but 2 capsules provide 120 mg of alpha-linolenic acid, a precursor of DHA that is also found in gram quantities in the usual American diet. Bottles are marked with an expiration date. Other fatty acids found in the capsules are routinely found in the diets of US women. All capsule bottles are sent directly to the University of Kansas Medical Center Investigational Pharmacy, which maintain packing receipts for study products.

D.3.2. Study randomization.

A study randomization schedule is generated by the Investigational Pharmacy, and each capsule bottle is labeled with the patient's name and unique study number. Only the Investigational Pharmacy and the Data Safety Monitoring Board (DSMB) has access to the individual assignments until the final data (i.e., the 18-month assessment, as this represents the end of the original RCT) are cleaned and locked pending statistical analysis. The medical monitor has the authority to request the assignment of individuals from the Investigational Pharmacy if it is needed for ongoing safety monitoring.

D.3.3. Study product records

The Investigational Pharmacy mails capsules to the subjects each month; the bottles and any remaining capsules from the previous month are returned by mail to the study coordinator, who transfers the envelope containing the bottle unopened to the Investigational Pharmacy, the capsules remaining counted, the number remaining recorded and destroyed. Records of capsules mailed to and received back from subjects and destroyed are also maintained there. The PI, the study site staff, and participants do not know which capsules are being consumed by each patient. The Investigational Pharmacy informs the PI and staff if an individual person returns more than 15 capsules in a given month, and study personnel then contact the subject by telephone to determine if there are any problems with the capsules, and encourage compliance as appropriate.

D.4. Data collection and monitoring

The study is being conducted in accordance with Good Clinical Practices (GCP) (ICH Guidelines, 2002) and the appropriate regulatory requirement(s). Essential clinical documents are maintained to demonstrate the validity of the study and the integrity of the data collected. One hundred percent of all primary outcome data and safety results and a proportion of other data collected are being monitored by an external data monitor before data entry. The proportion is determined by the data monitoring group, based on the actual incidence of errors observed in data entry. Additional data checks are made after data entry by the project statistician, Dr. Byron Gajewski, using established procedures for identifying potential errors. Master files are established at the beginning of the study, maintained for the duration of the trial, and retained according to the appropriate regulations.

A case report form (CRF, Appendix D) is completed for each study patient enrolled. The PI is entrusted with the responsibility to ensure the accuracy, completeness, legibility and timeliness of the data reported in the patient's CRF. Source documentation supporting the case report form data indicates the patient's participation in the trial and document the dates and details of study procedures, adverse events and patient status.

Case report forms are filled out with a black ball-point pen or typed; corrections to the form do not obscure the original entry. Each correction, addition or deletion is initialed and dated by the investigator or investigator's designated representative. The PI signs and dates the Investigator's Statement at the end of the CRF to endorse the recorded data. The PI maintains all study records according to GCPs. Records are retained for at least two years after the study in completed by the site.

D.5. Adverse events and safety monitoring

D.5.1. Definitions

An adverse event (AE) is any reaction, side effect or other undesirable event that occurs in conjunction with the use of the test product, whether or not the event is considered related to the test product. This includes new and worsening signs and symptoms of underlying or emerging disease or any patient complaint. Signs and symptoms considered normal for pregnant or delivering patients (headache, lower back pain or backache, abdominal pain, abdominal cramps and nausea)
are considered to be expected adverse events. Serious adverse event (SAE) include death, a life-threatening event, inpatient hospitalization or prolonging of an existing hospitalization, a persistent or significant disability/incapacity, or a congenital anomaly/birth defect.

Important medical events may be considered an SAE when (based upon appropriate medical judgment) they jeopardize the patient and may require medical or surgical intervention to prevent one of the outcomes listed above. Definitions of mild, moderate, and severe AEs are as described in the original protocol, as are the categorization of AEs as expected or unexpected and not related, remote, possible, probable, or definite in terms of their relationship to the product. SAEs are reported to the University of Kansas Medical Center IRB, the University of Missouri, Kansas City Adult IRB, the medical monitor (Dr. Michael Georgieff) the DSMB, and Martek Bioscience when discovered.

D.5.2. Safety monitoring.

A KUMC DSMB and consultants (one obstetrician, one neonatologist and one statistician) are in charge of safety monitoring a do twice yearly reviews. The consultant neonatologist (Dr. Georgieff) also acts as the medical monitor.

D.5.3. Study evaluations

The incidence of adverse experiences serves as the primary safety endpoint. Adverse event(s) will be collected as spontaneous reports by the patient for the duration of study participation. Adverse events will be reported during hospitalization for the infant delivery and any other pre-delivery hospitalizations (*i.e.*, for pre-term labor). At delivery, adverse events will be collected until hospital discharge for the study patient and her infant(s).

D.5.4. Efficacy endpoints.

The primary efficacy endpoints for *pregnancy outcomes* are: gestational age of the infant at delivery (days), birth weight of the infant at delivery (grams), length of the infant at delivery (cm), incidence of pre-term delivery (<37 weeks), and prevalence of low infant birth weight (<2,500 grams). The secondary efficacy endpoints is incidence of pre-eclampsia or eclampsia and gestational diabetes.

D.6. Determination of DHA Levels, Anthropometrics, Diet Histories and Medical Records

Maternal DHA levels are determined by blood draws at baseline and at delivery in the KUDOS trial. Infant DHA levels are determined from cord blood samples and by blood draw at 4 months of age. These data will be made available for use in the proposed study analyses. In addition, background diet DHA will be determined from 24-hour diet recalls conducted at each visit. The 24-hour recall method is easy to administer and minimizes subject burden. Multiple 24-hour dietary recalls have been shown to represent usual dietary intakes (Smiciklas-Wright, 1995) and have been validated in infants and preschool children (Klesges et al., 1987; Dop et al., 1994; Horst et al., 1988). They have been used as the "gold standard" for developing new methods such as food frequency questionnaires for infants/toddlers (Blum et al., 1999; Parish et al., 2003).

Personnel who will collect the diet recall information will be trained by Dr. Debra Sullivan to conduct standardized, structured interviews using neutral probing questions (Tillotson et al.1986). Dr. Sullivan has trained research staff to conduct multiple-pass 24-hour recalls from adults and children in numerous studies (DIHDH49181, DK58385, DK61489, Dairy Management, Inc., Sunflower Foundation, and our ongoing study of postnatal DHA and development). Intra-class correlations for dietary recalls among the research staff she supervises is consistently greater than 0.97. Parents/caregivers have been documented to be accurate reporters of their young children's dietary intake (Klesges et al., 1987; Basch et al., 1990). To promote accurate estimation of portion sizes, interviewers will use three-dimensional food models. Use of neutral probing questions and food models has been documented to decrease errors in dietary recalls (Gibson, 1990). Nutrient intake (including DHA) will be determined using the Nutrition Data System for Research (version 5.0_35, University of Minnesota, Minneapolis, MN) software. All interviewers will undergo two days of training by Dr. Sullivan on standardized dietary interview and computer coding methodology. After the training, they will be required to complete ten 24-hour dietary recalls on non-study subjects and directly enter the recalls into the nutrient database. The recalls will be evaluated according to a published dietary recall documentation checklist (Tillotson et al., 1986). An error rate of less than 6% on the recall documentation checklist and the computer coding will be required before interviewers can begin interviewing study subjects. During the study, all dietary recalls will be evaluated by the recall documentation checklist before entry into the study database. Any recall with greater than 6% error will be eliminated and another subject recall obtained.

Weight and length will be obtained at 6 mo intervals from 2- 6 y by a registered dietitian trained to do these standardized assessments. Birth weight and length will be obtained from the child's birth records. Body weight is recorded to the nearest 0.1 kg on an electronic scale with the child wearing light clothing (and dry diaper, if relevant). Statue is measured without shoes to the nearest 0.1 cm on a stadiometer from 2-6 y.

Starting with the 4-year visit and for every visit thereafter, children's blood pressure and heart rate will be assessed. Children are wheeled on a rolling chair from the testing room down the hall to the anthropometrics room. We will measure child BP using an automated system (GE Carescape V100) and employ the cuff size recommended for the child's arm circumference. BP is measured in triplicate when the child has been sitting for 5 minutes. Blood pressure is taken 3 times at each age and results averaged.

As part of each visit, parents will be asked to provide information about the health of their child since the last visit, including any illnesses. We will also obtain medical records following yearly visits (2, 3, 4, 5 and 6 years of age) to assess illnesses.

D.7. New Postnatal Behavioral Outcomes

The testing schedule and list of variables collected in the original RCT are listed in tables in section B.3. of the Background and Significance section. Collection and processing of biological samples and details concerning isolation of phospholipids are described in the original protocol.

The fundamental purpose of this supplement is the addition of a series of measures to this project that follow infants beyond 18 months. We have previously provided a theoretical rationale for the inclusion of each of these types of measures in Section B.4.1. In the following paragraphs, we outline the specific measures to be used, the information they provide, and the ages of assessment.

D.7.1. Higher-Order Cognition Measures

These measures represent the more complex types of cognitive function that emerge during the late toddler and early preschool periods. It is worth noting that we have included all of these tasks in other prior observational or experimental studies in our laboratory with children of the ages proposed. As such, our laboratory has extensive experience with the administration of these tasks, the coding schemes that they require, the time necessary to run them, and the data that they generate.

D.7.1.1. Explicit Memory Tasks (2 and 2.5 year visits; 20 min for assessment at each age). Explicit memory abilities at these ages is typically assessed using elicited-imitation tasks. In these tasks, children are presented with objects that are combined in a sequenced order to produce a product and final behavioral action or response (Bauer, 2000). The tasks proposed here have been used in our laboratory for several years now, and involve four actions, which the literature (e.g., Wenner & Bauer, 1999) suggests is appropriate for these ages. The tasks conform to a typical script: the child is given objects for the task and is allowed to manipulate them for some time (here, 2 minutes) so that the novelty of the items does not interfere with the performance of the task at hand. Then, the experimenter demonstrates the desired sequence for the child twice. The child is then asked to repeat the sequence immediately, after a 30 minute delay, or at a 6-month delay (e.g., the task assessed at 24 months is reassessed at 30 months). The child is given a 2-minute window to reconstruct the steps and repeat the task. Two novel events are assessed at each of the visits; as noted above, the 2.5 year visit includes a brief assessment of the 2 year sequences as well. The child's performance is quantified by coding the number of desired actions produced (there are 4 possible actions) and the number of desired pairs of actions produced *in the correct order* (this has a maximum score of 3). The latter score controls for the child's repetition of an action simply as a function of trial and error.

D.7.1.2. Spatial Memory Tasks. (2 and 2.5 year visits; 12 min for assessment at each age) Participants' spatial memory is assessed in a task similar to the "sandbox" task that has been used successfully in the past with young children (Spencer et al., 2001). Here, small plastic toys hidden are hidden in a 60" long x 16" wide x 8" deep box filled with dry lentils. The task begins with three practice trials in which the toy is partially buried and found by the child. To start the actual task, the toy is completely buried (with the child watching) at one location (Location A). The child's attention is distracted and held away from the display for a consistent interval by clapping and counting to 3. The child is then permitted to search for the buried object. After 3 successive retrievals from the first location (A), the toy is buried at a different location (B) for two successive trials. After a 10-second delay (clapping and counting), the child is allowed to search. After the child retrieves the toy the second time, the protocol is repeated by then hiding/burying the object at the original location (A). During testing, researchers remove cues to the location of the object by smoothing the substrate surface. The task is adjusted in difficulty at the two ages by moving the two hiding locations closer together at the older ages: at the 2-year visit, the locations are separated by 12" and then by 9" at the 2.5 year session. A camera positioned above the box records the child's responses and is facilitated by a tape measure tacked along the edge of the box. The primary dependent variable (i.e., accuracy of spatial memory) is the distance of the participant's first touch of the substrate surface from the actual location of the stimulus.

D.7.1.3. Distractibility (2 and 2.5 year sessions; 15 min for assessment at each age). Here, the child is given an object to play with that presents a task for the child to accomplish or a problem to solve. While executing the task or problem, we attempt to distract the child by activating a television clip in the child's peripheral vision. These tasks have been used in our laboratory since 2002; attentional performance here is related to inhibition, planning, memory, and attention span, and are linked directly with the frontal brain functions (Colombo &

Cheatham, 2006). In addition, they have previously been shown to be sensitive to DHA status (Colombo et al., 2004a; Kannass, Colombo, & Carlson, 2008). This session employs four 3-minute periods in which the child is given a different interesting and developmentally appropriate toy (e.g., building blocks, puzzles). Videotaped distractors are presented on a 26” television monitor placed at a 45° angle and 1m in distance from the child. The distractors are separated by random lengths of time (varying from 5 to 25 seconds) and are composed of both the visual and auditory tracks of Sesame Street clips. Videotapes are coded for three variables: (a) the proportion of times the child turns to the distractor, (b) the latency to turn, and (c) the length of time before returning attention to block/puzzle with which the child was originally playing. In addition, we code for the quality of participants' attention (“focused” versus “casual”; see, e.g., Ruff & Lawson, 1990) based on the quality of motor actions and facial expression, as better performance on this distraction task is typically observed under conditions of a more focused attentional state.

D.7.1.4. Counting Tasks (2, 2.5, 3.5, 4.5, and 5.5 year sessions; 15 min for assessment at each age). Several tasks will be used to assess the children's concept of number and counting ability.

Simple Count. Children will be asked to count on their own, without the aid of any objects. If a child pauses or loses his/her spot, the experimenter will prompt with the next number to see if the child can pick up where he/she left off. The dependent measure is the highest number reached with one or fewer prompts.

Counting Alone/Counting with Experimenter. For these tasks the children will be shown numerous blocks and a container. The children will first be asked to count the blocks while the experimenter puts them into the container. Next the children will be asked to count the blocks while they put them into the container themselves. If a child pauses or loses his/her spot, the experimenter will prompt with the next number to see if the child can pick up where he/she left off. The dependent measure is the highest number reached with one or fewer prompts for each task.

More/Less. Children will be shown a Bear puppet and a Dragon puppet. It will be explained that they each have different numbers of apples and we want to know who has more apples. Ten trials will be administered where a card with a different number of apples will be placed in front of each puppet. The order of the card pairs as well as which puppet is assigned more apples will be counterbalanced across children. The dependent measure is the number of correct trials.

Count/How Many. For this task the children will be presented with cards one at a time that have a different number of animals on them. They will first be asked to count how many animals there are. Once the children have finished counting, they will then be asked “How many are there?” A correct response will equal the number the child counted to, even if that number is incorrect. The dependent measure will be the highest number that children can count to and correctly answer the *how many* question.

Number Sort. For this task children are asked to sort a series of cards with different numbers of balls on them (1 – 9) into one of three boxes (small, medium, big). Each box has a picture of a car on it that varies in size across the boxes. For each trial, the experimenter points to the box with the big car and says “Big things go here”, points to the box with the medium car and says “Medium things go here”, and points to the box with the small car and says “Little things go here.” The experimenter then give the child a card, tells them how many balls are on it, and then asks them to put it into one of the three boxes. The dependent measures for this task are the sum and average of the number of balls in each box.

D.7.1.5. Rule-Learning/Inhibition Tasks Several age-appropriate tasks will be used to assess the children's use of working memory, inhibitory control, attentional flexibility, planning, and strategy use in performance on various complex tasks. The tasks proposed are also being used in our laboratory in a separate RCT, and the scientific literature (e.g., Carlson, 2005; Carlson & Moses, 2001) clearly shows that they are developmentally sensitive and age-appropriate.

Stroop and Stroop-Variant Tasks. (3, 3.5, 4, and 5 year visits: 10 min for assessment at each age) In these tasks, children are administered a task in the form of a game or problem, in which they are given a rule to respond in a manner that works against their natural tendencies. They are named after a famous effect in cognitive psychology (Stroop, 1935) in which subjects must name the ink color in which words are printed. The speed of naming in this type of task is slowed considerably if the words are names of colors, as the subject must inhibit reading the color word (i.e., their natural tendency to read) in order to produce the correct response based on the ink color. Similarly, in the proposed tasks, the child must keep a rule in mind and apply it to the situation, and inhibit a prepotent or natural tendency to produce the correct response.

In the first of these (Carlson & Moses, 2001), the child is presented with two colored rectangles – one yellow, one red (color knowledge is assessed prior to administration of the task). The child is told that, in this game, they should try and "trick" the researcher by "pointing to the wrong color" when the researcher names a color. That is, if the researcher says "Point to the yellow square," the child is supposed to point to the red one, or if the researcher says "Point to the red square," the child is supposed to point to the yellow one. If the child does not know color names, adjustments are made so that the color names are replaced with an object that is naturally associated with the color (e.g., "banana" for "yellow," "apple" for "red," etc.). Following two practice trials, 16 test trials are administered in which the lateral position and correct response is counterbalanced across trials and the order of correct response randomized. The primary dependent variable is the proportion of correct responses.

The second of these variants is a task commonly administered to preschoolers, the "Day/Night" form of the Stroop task (Gerstadt et al., 1994). Here, the child is presented with either a yellow moon with stars on a black background or a yellow sun on a white background. After assessing whether the child already possesses the moon-night and sun-day association, the child is then asked to work against that natural tendency by saying "day" when the moon is presented, and "night" when the sun is presented. After correct performance on 2 practice trials, 16 trials are presented, arranged as with the previous task. The primary dependent variable is the number correct.

Dimensional Change Card Sort (DCCS). (3, 3.5, 4, and 5 year visits: 10 min for assessment at each age) This test is based on the basic paradigms of discrimination learning (Kendler, 1979; Reese & Lipsitt, 1970), which was a focus of our laboratory's research program during the 1990s (Coldren & Colombo, 1994; Colombo et al., 1990). This particular task is administered according to the protocol published by Zelazo (2006), and has also been used in other nutritional studies, as well as in our research program on the development of attention in preschoolers (Coldren & Colombo, 2008). Essentially, participants are presented with stimuli that can be sorted on various dimensions (e.g., shape or color). They are asked to sort the stimuli on one dimension (thus providing a measure of performance on that ability) but then are asked to *reverse* (i.e., "switch") the rule. At that point, they must inhibit the previously activated rule in order to perform correctly under the new rules. For this particular task, children are presented a game in which they must sort 7 "red bunny" cards and 7 "blue car" cards into one of two boxes. One box is "labeled" with a red car and the other with a blue bunny. The child is asked to play either the "color game," with red bunnies sorted (face down) into the "red car" box; blue cars sorted (face down) into the "blue bunny" box; or they are asked to play the "shape game," with blue cars sorted (face down) into the "red car" box and red bunnies sorted (face down) into the "blue bunny" box. The protocol is to administer two practice trials, and then 6 trials with varying levels of support as needed (i.e., instructions may be repeated, or cards may be identified as a scaffold for the production of strategies) although the answers are never given to the child. If children perform 5 out of 6 trials correctly, the game is switched to the opposite (i.e., shape-to-color or color-to-shape). Should the child successfully negotiates the switch, the task would proceed with the "border version" of the task, where half of 12 sorting cards have a black border around the picture, and the child is asked to play "color game" when the card has a black border and "shape game" when it does not; here, there are 12 trials, but no game-switch phase. Correct responses are recorded for each phase, and performance is also qualitative characterized as 0 (fail pre-switch phase), 1 (pass pre-switch, fail post-switch), 2 (pass the pre- and post-switch phases, but fail border phase), and 3 (pass all phases).

Monkey Game/Modified Tower of Hanoi. (4, 5, and 6 year visits: 20 min for assessment at each age): This simplified version of the Tower of Hanoi paradigm (Klahr & Robinson, 1981; Welsh, 1991) assesses planning and strategy use. The modification allows use of the task in younger children, but even then, is not appropriate for children under the age of 4. The task is comprised of colored disks (monkeys) and 6 pegs (trees). Three pegs are for the participants; three for the researcher. The researcher relates a scripted story to the participant in which it is explained that the disks represent monkeys – daddy monkey (large disk), mommy monkey (medium disk), and baby monkey (small disk); the pegs represent their trees; and the table represents the river. It is explained that the monkeys in the participant's trees are copycats who always want to be the same as the monkeys in the researcher's trees. They are instructed to jump (move) the monkeys from tree to tree until they are in the same position as the researcher's monkeys. The following rules are related: 1) only one monkey can jump at a time, 2) monkeys can't swim, so they must never touch the river (table), and 3) bigger monkeys cannot sit on smaller monkeys because they will squish them. Legal and illegal moves are demonstrated; participants readily demonstrate understanding. The task is graded in difficulty starting with a 3-disk, 2-move problem progressing to a 7-move problem. The participants are given 6 trials with a maximum of 20 moves per trial to pass a problem. Pass is defined as solving the problem with as few moves as possible (i.e., solving a 3-move problem in 3 moves) twice in succession. Therefore, if no optimal solution has been reached by the 5th trial, no 6th trial is given. The primary dependent variable is a "processing efficiency score" based on which two trials the optimal solutions were achieved for each step (e.g., optimal solutions on trials 1 and 2 receive a score of 6, optimal solutions on trials 5 and 6 receive a score of 2).

Go/No-Go Task **.** (4.5 and 5.5 year visits, 30 min for electrode placement, 10 min for testing at each age) Event related potentials will be recorded while the participants complete a Go/NoGo task to measure inhibitory control and working memory. Data will be collected using individually placed electrodes (24 at 4.5 yrs and 34 at 5.5 yrs of age). The electrodes are placed using a small amount of paste that is easily washed out of the hair. Two additional electrodes are placed on the chest and record heart rate. The simplified, non-invasive methodology will facilitate participant retention: we are confident that the ERP protocol will not increase attrition. EOG data is collected from electrodes above and below the eye to control for blink artifacts. Data are referenced to Cz and then re-referenced to linked mastoids offline. Impedences are considered acceptable when they are less than 5 kΩ. Data are collected using SCAN 4.3 software connected to a SynAmps2 amplifier. Gains will be set at 50 for scalp leads and 5 for EOG electrodes. A 60-Hz notch filter will be utilized, and data will be sampled every 5 msec. The task will be presented on a computer screen by STIM2 software. Length of a trial is 3000 msec with stimulus presentation lasting 2000 msec followed by an inter-trial interval of 1000 msec. Participants are told that they will play a game in which they will see fish and sharks (developed by S. Wiebe in the laboratory of K. Espy, University of Nebraska, where they are collecting normative data). They are instructed to push the button if they see a fish, but not if they see a shark because the shark will tear their net. Feedback for a correct button press in response to a picture of a fish (Go) is a picture of the fish in a net. Feedback for incorrectly pressing the button in response to a picture of a shark (NoGo) is a picture of the shark in a torn net. Ten practice trials are given and then, the task proceeds with two blocks of data: 1) a block of 20 trials, all Go trials, and 2) a block of 80 trials, 25% NoGo trials pseudo-randomly presented. Trials will be rejected if they contain movement artifact greater than ±100 μV. After re-referencing and correcting for eye movement artifact, data will be visually inspected to determine the time intervals that include the N2 and the P3 as these are the components of interest. Outcome variables include latency to peak and peak amplitude. Data for each component are entered into separate Group (DHA, non-DHA) X Condition (Go, NoGo) X Lead (varies, e.g., midlines = Fz, Pz, Cz) repeated measures ANOVA using SAS Proc Mixed. Whereas the frontal N2 is thought to be indicative of cognitive control, we would expect that the DHA group would have a more mature waveform (decreased amplitude) relative to controls.

D.7.2. Literacy/Language Processing

D.7.2.1. The Test of Preschool Early Literacy (TOPEL; 3.5 year visit, 25-30 min**)** The TOPEL (Lonigan et al., 2007) is a test for children aged 3 to 5 years, and is designed to provide researchers with a reliable and valid

means of monitoring children's progress toward proficient literacy. It is among the best standardized and most widely-used evaluation of children's preliteracy levels in three domains: vocabulary, phonological awareness, and print knowledge (see also, Lonigan, 2006). The constructs measured by the TOPEL have been found to be manifest as a coherent factor that strongly predicts school achievement (Lemelin et al., 2007). The Print Knowledge subscale (36 items) measures alphabet knowledge and early knowledge about written language conventions and forms. Here, the child is asked to identify letters and written words, point to specific letters, identify letters associated with specific sounds, and say the sounds associated with specific letters. The Definitional Vocabulary subscale (35 items) measures single-word oral vocabulary and definitional vocabulary by having the child label a pictured object. Finally, the Phonological Awareness subscale (27 items) measures word elision and blending abilities. The child is asked to say a word, and then say what is left after dropping out specific sounds (elision) for the first 12 items; then to listen and separate sounds and combine them to form a word (blending) for the remaining 15 items. The test yields valid and reliable raw scores, standard scores, and percentiles. It has been normed on 842 preschool-aged children (3 to 5 years) sampled from representative areas across 12 US states. It is administered individually and takes 25-30 minutes to complete.

D.7.2.2. Peabody Picture Vocabulary Test III (PPVT-III; 5 year visit, 15 min) Receptive vocabulary will be assessed using the PPVT-III. The PPVT-III is a nationally standardized assessment of receptive vocabulary that has been shown to be fair with respect to cultural and economic status (Washington & Craig, 1999). In this task, participants are shown pages depicting 4 pictorial choices. For each page, the examiner says a word, and the participant indicates, either by pointing or by stating a reference number, which picture best matches the word. Administration is simple and straightforward. The PPVT-III yields a standard score normed with a mean of 100 and a standard deviation of fifteen.

D.7.2.3. Sentence Repetition Tasks (3, 3.5, and 4 year visits, 10 min at each age). Sentence repetition tasks have been used to assess language processing, memory for sentence structure, and other cognitive skills concomitant and contributing to language since the 1960s (Spreen & Benton, 1963). In these tasks, children are presented with sentences and are instructed to repeat the sentences back verbatim. The tests begin with simple sentences (e.g., "He was a good boy") but then become increasingly more difficult to repeat ("Where can he do what he wants?", "I asked him if he did it, and he said he didn't do it", etc.), both as a function of both length and syntactic complexity (Montgomery et al., 1978). These have been used successfully with preschoolers in the age range we are proposing (Carmichael & McDonald, 1984; Chafetz, 1994; Devescovi & Caselli, 2007; Montgomery et al., 1978). Repetitions are recorded and scored for omissions, additions, syntactic and articulation errors. Performance on this task is a strong predictor of later reading proficiency and fluency (Leong & Haines, 1978; Manis et al., 2004; Mann et al., 1984).

D.7.3. Adaptive Regulation

D.7.3.1. The Behavior Assessment System for Children, Version 2 (BASC-2): Parent Rating Scales (3, 4, and 5 year visits, 10-20 min)**.** The BASC (Reynolds & Kamphaus, 2006) is a widely-used instrument for comprehensive documentation of children's (ages 2 to 21) socioemotional behavior and adaptive regulation. We propose to use the Parent Rating Scales, which have been repeatedly proven to be reliable indicators of the child's adaptive function in many studies. The scales yield T scores and percentiles, for a general population and clinical populations. The system was originally designed for identifying children at risk for behavior problems due to self-regulation and socioemotional difficulties (see scales below), but is normed based on current U.S. Census population characteristics. The most recent revision of the BASC (BASC-2) was designed explicitly to be sensitive to developmental issues.

The Parent Rating Scales measure both adaptive and problem behaviors in the community and home setting. We will ask parents to complete forms for the preschool level (ages 2 to 5), which involves completing 134 items presented in a four-choice response format. The parent forms can be completed in 10-20 minutes, and requires only a 4th grade reading level to complete. The preschool forms provide evaluation of the child on twelve different subscales that measure adaptive and socioemotional function, many of which dimensions have been

established in either observational or experimental studies as being relevant outcomes for DHA (see section B.3): Adaptability, Aggression, Anxiety, Attention Problems, Depression, Hyperactivity, Withdrawl. The remaining subscales (Activities of Daily Living, Atypicality, Functional Communication, Social Skills, Somatization) provide basic adaptive, functional measures that can be seen as valuable as comprehensive measures of child outcomes as well.

D.7.4. Intelligence

D.7.4.2. The Weschler Preschool Primary Intelligence Scale-Third Edition (WPPSI-III, 3, 4, and 6 year visits: 35 min, 45 min, and 60 min respectively**).** The WPPSI-III (Weschler, 2002) is among the most widely-used and best-standardized tests of early intelligence available. The revisions undertaken for the most recent version of the test make it most appropriate for this age range and most desirable for the followup. It is appropriate for the ages to be tested for this followup (it is valid for ages 2.5 through 7.25 years), and the preschool form of the test has been streamlined to eliminate subtests that generally yield floor effects at young ages, reduce discriminability, and unnecessarily increase testing time; the preschool form of the test now takes only 25-35 minutes to administer at age 3, and 35-45 minutes at age 4. Major revisions in the materials also make the test more preschooler-friendly. We have used both this test and its major competitor (the Stanford-Binet) at these ages and have come to prefer this test because of these reasons.

At the earlier age-band (e.g., 3 year visit) level, the WPPSI-II is composed of four core subtests (Receptive Vocabulary, Information, Block Design, and Object Assembly). The first two subtests are combined to produce a Verbal IQ measure; the latter two to produce a Performance (i.e., Nonverbal) IQ. These two measures may then be combined to produce a Full-Scale (Composite) IQ. An additional supplemental subtest (Picture Naming, which is an expressive vocabulary measure) is also available. At the older age band (4- and 6-year visits), there are seven core subtests (Information, Vocabulary, Word Reasoning, Block Design, Matrix Reasoning, Picture Concepts, and Coding). The first three combine to form a Verbal IQ, the latter three combine for a Performance IQ. Coding can be added directly with the Verbal and Performance IQ when deriving a Full-Scale IQ score, or it can be combined with a Symbol Search supplemental subtest to derive a Processing Speed Quotient (PSQ). Four other supplemental subtests can be administered to substitute for the core tests, and two additional optional subtests (Receptive Vocabulary and Picture Naming) can be administered.

D.7.6. Summary Table and Visit Outline

The proposed additions for this supplement are outlined in the table below in an effort to summarize the longitudinal assessment schedule for 24-72 months of age. Note that we will continue to take anthropometric measures (height, weight, head circumference, etc.) at these visits as a matter of procedure.

In the table below, a number representing the number of minutes necessary to administer the measure appears in each cell where the assessment is taken. This provides an overall view of the schedule, as well as the overall time necessary to complete the visit. The time commitment is calculated separately for parents and children, and both are added for a total length. Note that the child and parent tasks can overlap in time (e.g., the parent can fill out the BASC while the child is engaged in other tasks), so the total time may represent a conservative (over)estimate of that commitment.

It should be noted that 24 month visits began in July of 2008, and so we will conduct some of these assessments, and absorb the costs of those assessments, until the decision regarding these supplemental funds has been made.

ASSESSMENT	VISIT AGE (months) 24	30	36	42	48	54	60	66	72
**Informed Consent**	10								
**Higher-Order Cognition**									
Spatial Memory	12	12							
Elicited Imitation	20	20							
Distractibility	15	15							
Counting	15	15		15		15		15	
Dimensional Change Card Sort			10	10	10		10		
Stroop Variants			10	10	10		10		
Modified Tower of Hanoi					20		20		20
Go/No-Go						40		40	
**Language and Pre-Literacy Assessment**									
Test of Preschool Early Literacy (TOPEL)				30					
Peabody Picture Vocabulary Test (PPVT-III)							15		
Sentence Repetition Tasks			15	15	15				
**Adaptive Regulation**									
Behavior Assessment System for Children (BASC-2) Parent Form			20		20		20		
**Intelligence**									
Weschler Primary Preschool Scale of Intelligence (WPPSI-III)			35		45				60
**Health & Diet**									
Anthropometrics	5	5	5	5	5	5	5	5	5
24-Hour Diet Recall	15	15	15	15	15	15	15	15	15
6-Month Illness Report	5	5	5	5	5	5	5	5	5
Blood Pressure/Heart Rate					5	5	5	5	5
Medical Records Requested	0		0		0		0		0
Child Mean Time Testing (min)	62	62	70	80	105	60	60	60	85
Parent Mean Time Testing/Forms (min)	35	25	45	25	45	25	45	25	25
**Total Session Length (min)**	**97**	**87**	**115**	**105**	**150**	**85**	**105**	**85**	**110**

D.9. Statistical methods

D.9.1. Descriptive/Preliminary statistics. Preliminary analyses will be initiated as they are for the original RCT. Simple descriptive statistics (means/standard deviations or frequencies/percentages) will be tabulated for all outcome and explanatory variables. Possible disparities between the treatment groups with respect to each continuous explanatory variable will be assessed through two-group *t*-tests. Disparities between the treatment groups with respect to dichotomous explanatory variables will be assessed through Fisher's exact test. Scatterplots of all outcome variables versus capsule intake will be produced separately for each treatment group. Correlations between outcome variables and explanatory variables will be tabulated.

D.9.2. Predictions and Analyses. The primary analyses will be relatively straightforward examinations of differences between placebo and active supplement groups. For variables that are repeated over time, this will take the form of mixed-model analyses involving factors of Visit (i.e., Age) and Group (Placebo vs. Supplement), as such analyses use all available longitudinal data. We hypothesize benefits across all variables for infants whose mothers were prenatally supplemented: better memory and advanced cognitive performance, better

preliteracy and language processing performance (i.e., higher TOPEL scores and fewer errors of all types on sentence repetition), and higher IQ (although analyses will also be conducted on IQ subtests, such as Verbal vs. Performance IQ to determine whether differences seen on these two subscales in Birch et al., 2007 might be replicable). We predict fewer problems identified in the BASC-2 and better scores on all domains there overall.

D.9.3 Power/sample size. As noted above, the current retention rate of 96% that we are seeing within the RCT to 18 months is much higher than the 50% we proposed in the original application; the goal there was to retain a sample of 100. Our attrition right now suggests an ending sample size of between 190 and 200, which would essentially doubles the power we originally projected for a followup, and provides power well beyond that which we originally proposed (Cohen, 1988, 1992). Even if our loss rate quintuples to 20%, we will retain a sample of 160, far surpassing our original power projections. As many of the measures we are currently proposing have not been previously employed in studies of DHA supplementation, we have no indication of possible effect sizes. The exception, however, for a reasonably comparable (higher) does of DHA is for IQ. The effect sizes (Cohen's *d*) comparing supplemented versus unsupplemented infants presented in Birch et al's (2007) study for IQ were .38 for unsupplemented vs. DHA and .49 for unsupplemented vs. DHA+ARA. If the effect sizes seen in our study are close to these, this presents no issues for power for this extension of the RCT, even at the ages we propose for this supplement.

E. HUMAN SUBJECTS

The University of Kansas Medical Center Human Subjects Committee (KUMC-HSC)and the University of Missouri, Kansas City IRBs and the Truman Medical Center Privacy Board have reviewed and approved the procedures and protocols for the original RCT study prior to implementation. The KUMC-HSC is currently reviewing the proposal for this additional longitudinal testing.

Parents will be re-consented at the 24 month visit to cover the 24-48 month assessment period. Consent forms and HIPPA disclosure information per IRBs will be given to all subjects and their signatures' witnessed. The consent forms will include a description of the study, nature of the data collection, the potential benefits and adverse reactions anticipated. The subjects are reassured that their usual care would not be changed, withdrawn, or reduced if they chose to withdraw from the study at any time. The research team personnel will abide by all tenets of the University confidentiality policies, as well as the Privacy Protection for Research Subjects. All research staff will remain current in their NIH required Human Subjects protection and HIPAA certification.

In order to maintain patient privacy, all case report forms, study reports and communications are identified by initials and the assigned patient number. Data monitors and auditors from the IRBs, and regulatory authorities will have access to the patient's original medical records for verification of data gathered on the case report forms and to audit the data collection process. Subjects are made aware of persons who may see their protected health information in the informed consent document and may choose not to enroll in the study based on the information provided them in accord with 2003 HIPAA regulations. The patient's confidentiality is maintained and is not made publicly available to the extent permitted by the applicable laws and regulations.

The PI will conduct the trial in compliance with the protocol given approval by the appropriate IRBs (the HSC at the Lawrence campus, which covers Dr. Colombo's activities, has a reciprocality agreement with the KUMC HSC). Any changes to the protocol will require written approval from these committees prior to implementation, except when the modification is needed to eliminate an immediate hazard(s) to patients or if the change(s) involves only logistical or administrative aspects of the trial. Any departures from the protocol will be fully documented in the case report form and source documentation.

E.1. Risks to the subjects

All information provided in this section pertains to the University of Kansas

E.1.1. Human subjects' involvement and characteristics. The study population will be 24-48-month-old children who have been enrolled in the original RCT covered by this application. As noted elsewhere, 350 pregnant women will be enrolled as part of that RCT, the majority of subjects (about 75%) are expected to be of African descent. Subjects not of African descent were divided among Hispanic, European-American and Asian descent. Inclusion and exclusion criteria are described elsewhere in the application narrative, and are not repeated here, as they apply to enrollment in the original RCT; the current supplement seeks to extend the longitudinal schedule for those enrollees, to whom the criteria have already been applied.

E.1.2. Sources of materials. All data will be gathered for the explicit purposes of this study using procedures to ensure confidentiality. All data will be identified by a code number only. A list that links the assigned code number to the subjects' name will be kept separately in a locked file. All subjects will be encouraged to contact the HSC with any concerns about the study.

E.1.3. Potential risks. No appreciable risk of physical, psychological, social, legal or other harm is expected from this additional testing. It is possible that the standardized testing will identify individual children at risk for developmental disability and delay. When that occurs, we collaborate with parents and determine whether they desire referral to a county agency or to a clinic within KUMC, and determine which (if any) records from the study they might want released. No release of information occurs without parent agreement and written consent.

E.2. Adequacy of protection against risks

E.2.1. Patient recruitment and informed consent. All recruitment, consent and data forms for the study proposal will be submitted to the IRBs of both Universities involved prior to any enrollment. Informed consent will be obtained by a trained research team member who has completed the NIH-approved Human Subjects' protection certification. Consent includes the standard elements: a study description, the potential risks, benefits and options for non-participation. Prior to proceeding with enrollment, personnel will explicitly ask the subjects to confirm that they know components of the study involve taking a nutritional supplement for the remainder of their pregnancy. All subjects will be informed they are free to withdraw from the study without changes in their usual care. Consent will be documented as a signed form and will be kept in a locked file at the study office. The study will be conducted in accordance with ethical principles founded in the Declaration of Helsinki and the Belmont Report. The IRBs will review all appropriate study documentation in order to safeguard the rights, safety and well-being of the subjects. The protocol, informed consent, written information given to the subjects, safety updates, annual progress reports, and any revisions to these documents will be provided to the IRBs by the investigator. The method of obtaining and documenting the informed consent and the contents of the consent will comply with all applicable regulatory requirement(s).

E.2.2. Protection against risk: No appreciable risk of physical or mental harm is expected to result from the additional longitudinal protocol. Carlson has been conducting clinical trials continuously since 1978 in this or a similar population; Colombo has conducted longitudinal research continuously since 1984. Subjects who choose to withdraw from the study will be reassured if they indicate the desire to withdraw.

Subjects are protected against the risk of breaking confidentiality by decoupling of names from databases. The CRF will make use of a subject code number only, and informed consent forms that include the subject's signature will be stored separately in locked file cabinets. For ensuring confidentiality, these are generally acknowledged to be the best methods known for ensuring that names are not associated with data. Only selected research staff will have access to the subjects' data.

Subjects' informed consent includes the HIPAA compliance documentation approved by the KUMC and Lawrence HSCs. The Research team personnel will abide by all tenets of the University confidentiality policies as well as the Privacy Protection for Research Subjects.

E.3. Potential benefits of the proposed research to the subjects and others

This study could result in outcomes which contribute to new knowledge about the effects of prenatal nutritional intake of pregnant women on children.

E.4. Importance of the knowledge to be gained

The significance of this work lies with the evaluation of the long-term effects of prenatal supplementation on meaningful variables in the preschool period. We hypothesize that prenatal supplementation may accrue long-term benefits to the offspring of women; the demonstration that prenatal nutrition affects complex cognition, preliteracy/language measures, adjustment/adaptive measures, and intelligence up to 4 years from the point of supplementation will be extraordinary.

E.5. Women and minority inclusion in research

Both boys and girls will be included in the study. The majority are of African-American descent, as a function of the locales in which the original enrollment was executed.

E6. Inclusion of children in clinical research

The primary participants in the study are children who were enrolled in the original RCT, and so the inclusion of minorities is a fundamental characteristic of the proposed study.

E.7. Special populations

Not applicable.

E.8. Data safety and monitoring plan

The data safety plan for the original RCT continues in place, although the additional longitudinal measures are not considered to be part of the RCT.

E.9. Anticipated areas of difficulty.

None.

F. VERTEBRATE ANIMALS

Not applicable

G. LITERATURE CITED

Agostoni C, Trojan S, Bellu R, Riva E, Bruzzese MG, & Giovannini M. (1995). Neurodevelopmental quotient of healthy term infants at 4 months and feeding practice: the role of long-chain polyunsaturated fatty acids. *Pediatr. Res. 38*, 262–266.

Agostoni C, Trojan S, Bellu R, Riva E, Bruzzese MG, & Giovannini M (1997). Developmental quotient at 24 months and fatty acid composition of diet in early infancy: a follow up study, *Arch. Dis. Child. 76*, 421–424.

Auestad N, Montalto MB, Hall RT, Fitzgerald KM, Wheeler RE, Connor WE, Neuringer M, Connor SL, Taylor JA, & Hartmann EE (1997). Visual acuity, erythrocyte fatty acid composition, and growth in term infants fed formulas with long chain polyunsaturated fatty acids for one year, *Pediatr. Res. 41*, 1–10.

Auestad N, Scott DT, Janowsky JS, Jacobsen C, Carroll RE, Montalto MB, Halter R, Qiu W, Jacobs JR, Connor WE, Connor SL, Taylor JA, Neuringer M, Fitzgerald KM, Hall RT. (2003). Visual, cognitive, and language assessments at 39 months: a follow-up study of children fed formulas containing long-chain polyunsaturated fatty acids to 1 year of age. *Pediatrics, 112*, e177-83.

Baguma-Nibasheka M, Brenna JT, Nathanielez PW. (1999). Delay of preterm delivery in sheep by omega-3 long-chain polyunsaturates. *Biol Reprod , 60*, 698-701.

Bakker EC, Hornstra G, Blanco CE, Vles JSH. (2007). Relationship between long-chain polyunsaturated fatty acids at birth and motor function at 7 years of age. *Eur J Clin Nut*, advance online publication, 19 Dec. 2007;doi:10.1038/sj.ejcn.1602971).

Bakker EC, van Houwelingen AC, & Hornstra G (1999). Early nutrition, essential fatty acid status and visual acuity of term infants at 7 months of age, *Eur. J. Clin. Nutr. 53*, 872–879.

Barkley RA (1997). Behavioral inhibition, sustained attention, and executive functions: Constructing a unifying theory of ADHD. *Psychol Bull,121,* 65-94.

Barkley RA. (2000). Genetics of childhood disorders: XVII. ADHD, Part I: The executive functions and ADHD. *J Amer Acad Child & Adol Psychiatry, 39,* 1064-1068.

Bauer PJ (2004). Getting explicit memory off the ground: Steps toward construction of a neurodevelopmental account of changes in the first two years of life. *Devel Rev, 24,* 347-373

Bell MA, Wolfe CD (2007). Changes in brain functioning from infancy to early childhood: Evidence from EEG power and coherence during working memory tasks. *Devel Neuropsych, 31*, 21-38

Birch DG, Birch EE, Hoffman DR & Uauy, RD (1992a). Retinal development in very-low-birth-weight infants fed diets differing in omega-3 fatty acids. *Investig. Ophthalmol.Vis. Sci. 33*, 2365–2376.

Birch DG, Birch EE, Hoffman DR, & Uauy RD (1992b). Dietary essential fatty acid supply and visual acuity development. *Investig. Ophthalmol. Vis. Sci. 33*, 3242–3253.

Birch EE, Birch DG, Hoffman DR, Uauy RD. (1992c). Dietary essential fatty acid supply and visual acuity development. Invest. *Ophthalmol Vis Sci, 33,* 3242-3253.

Birch EE, Garfield S, Castañeda Y, Hughbanks-Wheaton D, Uauy R, Hoffman D. (2007). Visual acuity and cognitive outcomes at 4 years of age in a double-blind, randomized trial of long-chain polyunsaturated fatty acid-supplemented infant formula. *Early Hum Dev, 83,* 279-284.

Birch EE, Garfield S, Hoffman DR, Uauy R, & Birch DG (2000). A randomized controlled trial of early dietary supply of long-chain polyunsaturated fatty acids and mental development in term infants, *Dev. Med. Child Neurol. 42*, 174–181.

Birch EE, Hoffman DR, Uauy R, Birch DG, & Prestidge C (1998). Visual acuity and the essentiality of docosahexaenoic acid and arachidonic acid in the diet of term infants. *Pediatr. Res. 44*, 201–209.

Black MM, Holden EW (1995). Longitudinal intervention research in children's health and development. *J Clinl Child Psychol, 24*, 163-172

Blaga O, Anderson CJ, Shaddy DJ, Kannass KN Little T, & Colombo J (2008, under review). Structure and continuity of mental development during early childhood. *Intelligence.*

Booth AE, Waxman S. (2002). Object names and object functions serve as cues to categories for infants. *Devel Psychol, 38*, 948 – 957.

Bootsmiller BJ, Ribisl KM, Mowbray CT, Davidson WS, Walton MA, Herman SE. (1998). Methods of ensuring high follow-up rates: Lessons from a longitudinal study of dual diagnosed participants. *Subst Use Misuse, 33*, 2665-2685.

Buydens-Branchey L, Branchey M, Hibbeln JR. (2008). Associations between increases in plasma n-3 polyunsaturated fatty acids following supplementation and decreases in anger and anxiety in substance abusers. *Prog Neuropsychopharmacol Biol Psychiatry, 32,* 568-575.

Capaldi D, Patterson GR. (1987). An approach to the problem of recruitment and retention rates for longitudinal research. *Behav Assess, 9*, 169-177.

Carlson SE, Ford AJ, Werkman SH, Peeples JM, & Koo WW (1996a). Visual acuity and fatty acid status of term infants fed human milk and formulas with and without docosahexaenoate and arachidonate from egg yolk lecithin. *Pediatr.Res. 39,* 882–888.

Carlson SE, Werkman SH, & Tolley EA (1996b). Effect of long-chain n-3 fatty acid supplementation on visual acuity and growth of preterm infants with and without bronchopulmonary dysplasia. *Am. J. Clin. Nutr. 63*, 687–697.

Carlson SE, Werkman SH, Peeples JM, & Wilson WM. (1994). Long-chain fatty acids and early visual and cognitive development of preterm infants. *Eur. J. Clin Nutr. 48 (Suppl.2)*, S27–S30.

Carlson SE, Werkman SH, Rhodes PG, & Tolley EA (1993). Visual-acuity development in healthy preterm infants: effect of marine-oil supplementation. *Am. J. Clin. Nutr. 58*, 35–42.

Carlson SE, Werkman SH. (1996c). A randomized trial of visual attention of preterm infants fed docosahexaenoic acid until 2 months. *Lipids, 31*, 85-90.
Carlson SM, Moses LJ. (2001). Individual differences in inhibitory control and children's theory of mind. *Child Devel*, *72*, 1032-1053.
Carlson SM. (2005). Developmentally sensitive measures of executive function in preschool children. *Devel Neuropsychol*, *28*, 595-616.
Carmichael JA, MacDonald JW (1984). Developmental norms for the Sentence Repetition Test. *J Consult Clin Psychol, 53,* 476-477
Carrie I, Clement M, DeJavel D, Frances H, & Bourre JM (1999). Learning deficits in first generation of1 mice deficient in (n-3) polyunsaturated fatty acids do not result from visual alteration, *Neurosci. Lett. 266*, 69–72.
Casebolt, K., Colombo, J., & Chandler, J. (2002). Teachers' perceptions and the TOMI as predictors of visual/visual-motor skills in motorically impaired and non-impaired elementary school-aged children. *Phys Educator, 60,* 34-41.
Chafetz J (1994). The closed-class vocabulary as a closed set. *Appl Psycholing, 15*, 273-287
Cheatham CL, Colombo J, Carlson SE. (2008b, in press). Long chain fatty acids in the developing retina and brain. In DF Anderson, RA Polin, WW Fox (Eds.), *Fetal and neonatal physiology.* Philadelphia, PA: WB Saunders.
Cheatham CL, Colombo J, Carlson SE. (2008a). *The effect of feeding formula enriched with docosahexaenoic acid on the declarative memory abilities of 10-month-old infants.* International Society for the Study of Fatty Acids and Lipids, Kansas City MO.
Cheruku SR, Montgomery-Downs HE, Farkas SL, Thoman EB, Lammi-Keefe CJ. (2002). Higher maternal plasma docosahexaenoic acid during pregnancy is associated with more mature neonatal sleep-state patterning. *Am J Clin Nutr, 76*, 608-613.
Clandinin MT, Chappell JE, Leong S, Heim T, Swyer PR, chance GW. (1980). Intrauterine fatty acid accretion rates in human brain: implications for fatty acid requirements. *Early Human Dev, 4*, 121-129.
Cohen J (1988). *Statistical power analysis for the behavioral sciences.* Hillsdale, NJ: Erlbaum.
Cohen J. (1992). A power primer. *Psychol Bull, 112*, 155-159.
Coldren JT, Colombo J. (1994). The nature and processes of preverbal learning: Implications from nine-month-old infants' discrimination problem solving. *Mon Soc Res Child Devel*, *59*(4, #249).
Coldren JT, Colombo J. (2008, under review). Attention as a cueing function during kindergarten children's dimensional change task performance. *Infant and Child Development.*
Colombo J (2001a). The development of visual attention in infancy. *Annual Review of Psychology*, *52*, 337-367.
Colombo J (2002). Infant attention grows up: The emergence of a developmental cognitive neuroscience perspective. *Curr Dir Psychol Sci*, *11,* 196-199.
Colombo J, Carlson SE, Cheatham CL, Kannass KN, Gustafson KM, Schmeidler T. (2008c) *Dietary supplementation with DHA in infancy lowers heart rate and increases sustained attention.* International Society for the Study of Fatty Acids and Lipids, Kansas City MO.
Colombo J, Cheatham CL (2006). The emergence of endogenous attention in infancy and early childhood. In R Kail (Ed.), *Advances in child development and behavior* (pp. 283-322). New York: Elsevier.
Colombo J, Kannass KN, Shaddy DJ, Maikranz JM, Kundurthi S, Anderson CJ, Blaga O ,& Carlson SE. (2004). Maternal DHA and the development of attention in infancy and toddlerhood. *Child Devel*, *75,* 1254-1267.
Colombo J, Mitchell DW, Coldren JT, Atwater JD. (1990). Discrimination learning during the first year of life: Stimulus and positional cues. *J Exp Psychol: Learn Mem Cog, 16,* 98-109.
Colombo J, Richman WA, Shaddy DJ, Maikranz JM, & Blaga O (2004b). Developmental course of visual habituation and preschool cognitive and language outcome. *Infancy*, *5,* 1-38.
Colombo J, Shaddy DJ, Blaga OM, Anderson CJ, Kannass KN (2008a, in press) High cognitive ability in infancy In F Horowitz, D Matthews (Eds) *Concepts of giftedness in developmental theory and research.* Washington DC: American Psychological Association Press
Colombo J, Shaddy DJ, Blaga OM, Anderson CJ, Kannass KN, Richman WA. (2008b, in press). Attentional predictors of vocabulary from infancy. In J Colombo, P McCardle, L Freund (Eds), *Infant pathways to language: Methods, models, and research directions*. Mahwah, NJ: Erlbaum.

Colombo J, Shaddy DJ, Richman WA, Maikranz JM, Blaga O (2004b) Developmental course of visual habituation and preschool cognitive and language outcome *Infancy, 5,* 1-38
Colombo J. (2001b). Recent advances in the assessment of infant cognition: Implications for LC-PUFA supplementation studies. *Lipids, 36,* 919-926.
Connor WE, Lowensohn R, Hatcher L. (1996). Increased docosahexaenoic acid levels in human newborn infants by administration of sardines and fish oil during pregnancy. *Lipids, 31*, S183-7.
D'Almeida A, Carter JP, Anatol A, Prost C. (1992). Effects of a combination of evening primrose oil (gamma linolenic acid) and fish oil (eicosapentaenoic + docosahexaenoic acid) versus magnesium, and versus placebo in preventing pre-eclampsia. *Womens' Health, 19*, 117-131.
Desimone R, Duncan J. (1995). Neural mechanisms of selective visual attention. *Ann Rev Neurosci, 18*, 193-222.
Devescovi A, Caselli MC (2007). Sentence repetition as a measure of early grammatical development in
Elias MF, Robbins MA. (1991). Where have all the subjects gone? Longitudinal studies of disease and cognitive function. In LM Collins, JL Horn (Eds.) *Best methods for the analysis of change: Recent advances, unanswered questions, future directions.* (pp. 264-275). Washington, DC, US: APA Press.
Ellickson PL, Bianca D, Schoeff DC. (1988). Containing attrition in school-based research: An innovative approach. *Eval Rev, 12*, 331-351.
Faldella G, Govoni M, Alessandroni R, Marchiani E, Salvioli GP, Biagi PL, & Spano C (1996). Visual evoked potentials and dietary long chain polyunsaturated fatty acids in preterm infants. *Arch. Dis. Child. 75,* F108–F112.
Farquharson J, Jamieson EC, Abbasi KA, Patrick WJ, Logan RW, Cockburn F. (1995). Effect of diet on the fatty acid composition of the major phospholipids of infant cerebral cortex. *Arch Dis Child, 72*, 198-203.
Farrington DP. (1991). Longitudinal research strategies: Advantages, problems, and prospects. *J Amer Acad Child Adol Psychiat, 30*, 369-374.
Fleischhauer FJ, Yan W-D, Fischell TA. (1993). Fish oil improves endothelium-dependent coronary vasodilation in heart transplant recipients. *J Am Coll Cardiol, 21*, 982-989
Forsyth JS, Willatts P, DiModugno MK, Varma S, & Colvin M (1998). Do long-chain polyunsaturated fatty acids influence infant cognitive behaviour? *Biochem. Soc. Trans,. 26*, 252–257.
Gerrard J, Popeski D, Ebbeling L, Brown P, Hornstra G. (1991). Dietary omega 3 fatty acids and gestational hypertension in the Inuit. *Arctic Med Res,* Suppl:763-7.
Gerstadt CL, Hong YJ, Diamond A. (1994). The relationship between cognition and action: performance of children 3 1/2-7 years old on a Stroop-like day-night test. *Cognition, 53*, 129-153.
Gibson RA & Makrides, M (1999). Polyunsaturated fatty acids and infant visual development: A critical appraisal of randomized clinical trials. *Lipids 34*, 179–184.
Gibson RA, Chen W, Makrides M. (2001). Randomized trials with polyunsaturated fatty acid interventions in preterm and term infants: functional and clinical outcomes. *Lipids, 36,* 873-883.
Given BA, Keilman LJ, Collins C, Given CW. (1990). Strategies to minimize attribution in longitudinal studies. *Nursing Res, 39*, 184-186.
Gopnik A, Meltzoff A. (1987). The development of categorization in the second year and its relation to other cognitive and linguistic developments. *Child Devel, 58,*1523-1531.
Goyette CH, Conners CK, Ulrich RF.(1978). Normative data on revised Conners Parent and Teacher Rating Scales. *J Abnorm Child Psychol, 6*, :221-236
Greiner RS, Moriguchi T, Hutton A, Slotnick BM, Salem N. (1999). Rats with low levels of brain docosahexaenoic acid show impaired performance in olfactory-based and spatial learning tasks. *Lipids, 34*, S239-243.
Hamazaki T, Hirayama S. (2004). The effect of docosahexaenoic acid-containing food administration on symptoms of attention-deficit/hyperactivity disorder-a placebo-controlled double-blind study. *Eur J Clin Nutr, 58,* 838.
Hamazaki T, Sawazaki S, Itomura M, Asaoka E, Nagao Y, Nishimura N. (1996). The effect of docosahexaenoic acid on aggression in young adults. A placebo-controlled double-blind study. *J Clin Invest, 97*, 1129–1133.

Hamazaki T, Sawazaki S, Nagao Y, Kuwamori T, Yazawa K, Mizushima Y, Kobayashi M. (1998). Docosahexaenoic acid does not affect aggression of normal volunteers under nonstressful conditions. A randomized, placebo-controlled, double-blind study. *Lipids, 33*, 663-667.

Hamazaki T, Sawazaki S, Nagasawa T, Nagao Y, Kanagawa Y, Yazawa K. (1999). Administration of docosahexaenoic acid influences behavior and plasma catecholamine levels at times of psychological stress. Lipids, 34, S33-S37.

Hamazaki T, Thienprasert A, Kheovichai K, Samuhaseneetoo S, Nagasawa T, Watanabe S. (2002). The effect of docosahexaenoic acid on aggression in elderly Thai subjects--a placebo-controlled double-blind study. *Nutr Neurosci, 5*, 37-41.

Hartsough CS, Babinski LM, Lambert NM. (1996). Tracking procedures and attrition containment in a long-term follow-up of a community-based ADHD sample. *J Child Psychol Psychiat Allied Discipl, 37*, 705-713.

Helland IB, Saugstad OD, Smith L, Saarem K, Solvoll K, Ganes T, Drevon CA. (2001). Similar effects on infants of n-3 and n-6 fatty acids supplementation to pregnant and lactating women. *Peds*, 108/5/e82 (http://www.pediatrics.org/cgi/content/full/108/5/e82)

Helland IB, Smith L, Saarem K, Saugstad OD, Drevon CA. (2003). Maternal supplementation with very long-chain n-3 fatty acids during pregnancy and lactation augments children's IQ at 4 years of age. *Pediatrics, 111*, e39-44.

Hibbeln JR, Ferguson TA, Blasbalg TL. (2006). Omega-3 fatty acid deficiencies in neurodevelopment, aggression and autonomic dysregulation: opportunities for intervention. *Int Rev Psychiat, 18*, 107-118.

Hirayama S, Hamazaki T, Terasawa K. (2004). Effect of docosahexaenoic acid-containing food administration on symptoms of attention-deficit/hyperactivity disorder - a placebo-controlled double-blind study. *Eur J Clin Nutr, 58*, 467-473.

Hough RL, Tarke H, Renker V, Shields P. (1996). Recruitment and retention of homeless mentally ill participants in research. *J Consult Clinical Psychol, 64*, 881-891.

ICH Guidelines 2002. Code of Federal Regulations, Title 21-*Good clinical practice (E6) and clinical safety data management* (E2A).

Innis SM, Akrabawi SS, Diersen-Schade DA, Dobson MV & Guy DG (1997). visual acuity and blood lipids in term infants fed human milk or formulae, *Lipids 32*, 63–72.

Innis SM, et al. (2002). Early language development and visual acuity are related to docosahexaenoic acid in breast-fed term infants. *93rd AOCS Annual Meeting Abstracts*, Montreal, Canada, p S73.

Innis SM, Friesen RW. (2008). Essential n-3 fatty acids in pregnant women and early visual acuity maturation in term infants. Am J Clin Nutr, 87, 548-557.

Innis SM, Gilley J, Werker J. (2001). Are human milk long-chain polyunsaturated fatty acids related to visual and neural development in breast-fed term infants? *J Pediatr, 139,* 532-538

Innis, SM (1992). Plasma and red blood cell fatty acid values as indexes of essential fatty acids in the developing organs of infants fed with milk or formulas. *J Pediatr, 120,* S78-86.

Iribarren C, Markovitz JH, Jacobs DR Jr, Schreiner PJ, Daviglus M, Hibbeln JR. (2004). Dietary intake of n-3, n-6 fatty acids and fish: relationship with hostility in young adults--the CARDIA study. *Eur J Clin Nutr, 58,* 24-31.

Isaev VA, Kaplan A, Kochetova AG, Platonova RD, & Ashmarin IP (2000). The complex of unsaturated fatty acids eikonol optimizes human cognitive activity, *Hum. Physiol. 26*, 210–215.

Italian. *Int J Lang Comm Dis, 42,* 187-208

Jacobson JL, Jacobson SW, Muckle G, Kaplan-Estrin M, Ayotte P, Dewailly E. (2008). Beneficial effects of a polyunsaturated fatty acid in infant development: evidence from the Inuit of Arctic *Quebec. J Pediatr, 52,* 356-364.

Jensen CL, et al (2004). Effects of maternal docosahexaenoic acid (DHA) supplementation on visual function and neurodevelopment of breast-fed infants. *Pediatr Res 49*:448A, 2001.

Jensen CL, Prager TC, Fraley JK, Chen H, Anderson RE, & Heird WC (1997). effect of dietary linoleic/alpha-linolenic acid ratio on growth and visual function of term infants, *J. Pediatr. 131*, 200–209.

Jensen CL, Prager TC, Zou Y, Fraley JK, Maude M, Anderson RE, Heird WC. (1998). Effects of maternal docosahexaenoic acid (DHA) supplementation on visual function and growth of breast-fed infants. *Pediatr Res, 43,* 262A.

Jensen MM, Skarsfeldt T, & Høy CE (1996). Correlation between level of (n-3) polyunsaturated fatty acids in brain phospholipids and learning ability in rats: a multiple generation study, *Biochim. Biophys. Acta 1300*, 203–209.

Julien M, Pomerleau A, Malcuit G, Rome-Flanders T. (1992). [The problem of recruitment and retention of a socioeconomically disadvantaged population in a longitudinal study]. *Science et Comportement*, *22*, 263-277.

Kane MJ, Engle RW (2002). The role of prefrontal cortex in working-memory capacity, executive attention, and general fluid intelligence: An individual-differences perspective. *Psychonomic Bull & Rev, 9,* 637-671.

Kannass, K.N., Colombo, J., & Carlson, S. E. (2008, under review). Maternal DHA levels and toddler free play attention. *Developmental Neuropsychology.*

Kendler T. (1979). Development of discrimination learning: A levels-of-functioning explanation. In H Reese (Ed.), *Advances in child development and behavior* (pp. 83-117). New York: Academic Press.

Kidd PM (2007). Omega-3 DHA and EPA for cognition, behavior, and mood: clinical findings and structural-functional synergies with cell membrane phospholipids. *Altern Med Rev, 12*, 207-227.

Krasevec JM, Jones PJ, Cabrera-Hernandex A, Mayer DL, Connor WE. (2002). Maternal and infant essential fatty acid status in Havana, Cuba. *Am J Clin Nutr, 76*, 834-844.

Lapillone A, Carlson SE. (2001). Polyunsaturated fatty acids and infant growth. *Lipids, 36,* 901-911.

Lemelin JP, Boivin M, Forget-Dubois N, Dionne G, Séguin JR, Brendgen M, Vitaro F, Tremblay RE, Pérusse D. (2007). The genetic-environmental etiology of cognitive school readiness and later academic achievement in early childhood. *Child Dev, 78*, 1855-1869.

Leong CK, Haines CF (1978). Beginning readers' analysis of words and sentences. *J Read Behav, 10,* 393-407.

Lindmark L, Clough P. (2007). A 5-month open study with long-chain polyunsaturated fatty acids in dyslexia. *J Med Food, 10,* 662-666.

Lonigan CJ. (2006). Development, assessment, and promotion of preliteracy skills. *Early Educ Devel, 17,* 91-114.

Lonigan CJ, Wagner RK, Torgesen JK. (2007). *The Test of Preschool Early Literacy.* Greenville, SC: Super Duper Publications.

Ma XH, Wu WX, Brenna JT, Nathanielsz PW. (2000). Maternal administration of long-chain n-3 polyunsaturates to the pregnant ewe in late gestation results in specific inhibition of prostaglandin h synthase (PGHS) 2, but not PGHS1 and oxytocin receptor mRNA in myometrium during betamethasone-induced labor. *J Soc Gynecol Invest, 7,* 233-237.

Makrides M, Neumann M, Simmer K, Pater J, & Gibson R (1995). Are long-chain polyunsaturated fatty acids essential nutrients in infancy? *Lancet, 345*, 1463–1468.

Makrides M, Neurman MA, Simmer K, & Gibson RA (2000). A critical appraisal of the role of dietary long-chain polyunsaturated fatty acids on neural indices of term infants: a randomized, controlled trial. *Pediatrics 105*, 32–38.

Makrides A, Neumann MA, Byard RW, Simmer K, Gibson RA. (1994). Fatty acid composition of brain, retina, and erythrocytes in breast- and formular-fed infants. *Am J Clin. Nutr, 60,* 189-194.

Malcolm CA, McCulloch DL, Montgomery C, Shepherd A, Weaver LT. (2003) Maternal docosahexaenoic acid supplementation during pregnancy and visual evoked potential development in term infants: a double blind, prospective, randomised trial. *Arch Dis Child Fetal Neonatal Ed, 88,* F383-90.

Manis FR, Lindsey KA, Bailey CE (2004). Development of reading in grades K-2 in Spanish-speaking English-language learners. *Lrn Disab Res Prac, 19*, 214-224.

Mann VA, Shankweiler D, Smith ST. (1984). The association between comprehension of spoken sentences and early reading ability: The role of phonetic representation. *J Child Lang, 11,* 627-643

Martinez M. (1991). Developmental profiles of polyunsaturated fatty acids in the brain of normal infants and patients with peroxisomal diseases: severe deficiency of docosahexaenoic acid in Zellweger's and pseudo-Zellweger's syndromes. *World Rev Nutr Diet, 66,* 87-102.

McCann JC, Ames BN (2005). Is docosahexaenoic acid, an n-3 long-chain polyunsaturated fatty acid, required for development of normal brain function? An overview of evidence from cognitive and behavioral tests in humans and animals. *Am J Clin Nutr, 82*, 281–295.

McNamara RK, Carlson SE. (2006). Role of omega-3 fatty acids in brain development and function: potential implications for the pathogenesis and prevention of psychopathology. *Prostaglandins Leukot Essent Fatty Acids*, *75*, 329-349.

Menard S. (1991). *Longitudinal research*. Thousand Oaks, CA: Sage.

Miller EK, Cohen JD. (2001). An integrative theory of prefrontal cortex function. *Ann Rev Neurosci, 24,* 167 – 202.

Montgomery C, Speake BK, Cameron A Sattar N, Weaver LT. (2003). Maternal docosahexaenoic acid supplementation and fetal accretion. *Br J Nut, 90*, 135-145.

Montgomery MM, Montgomery AA, Stephens MI (1978). Sentence repetition in preschoolers: Effects of length, complexity, and word familiarity. *J Psycholing Res, 7,* 435-452

Moore MK, Meltzoff AN (1999). New findings on object permanence: A developmental difference between two types of occlusion. *Brit J Devel Psychol, 17,* 623-644

Moriguchi T, Greiner RS, Salem N. (2000). Behavioral deficits associated with dietary induction of decreased brain docosahexaenoic acid concentration. *J Neurochem, 75*, 2563-2573.

Mozaffarian D. (2005) Does alpha-linolenic acid intake reduce the risk of coronary heart disease? A review of the evidence. *Altern Ther Health Med*, *11,*24-30.

Nair SSD, Leitch JW, Falconer J, Garg ML. (1997). Prevention of cardiac arrhythmia by dietary (n-3) polyunsaturated fatty acids and their mechanism of action. *J Nutr , 127*, 383-393.

Olsen SF, Hansen HS, Secher NJ, Jensen B, Sandstrom B. (1995). Gestation length and birth weight in relation to intake of marine n-3 fatty acids. *Br J Nutr, 73*, 397-404.

Olsen SF, Secher NJ, Tabor A, Weber T, Walker JJ, Gluud C. (2000). Randomised clinical trials of fish oil supplementation in high risk pregnancies. *Br J Obstet Gynecol, 107*, 382-395.

Olsen SF, Secher NJ. (2002). Low consumption of seafood early in pregnancy as a risk factor for preterm delivery: prospective cohort study. *Brit Med J , 324*, 447

Oskamp S, Mindick B, Berger D, Motta E. (1978) A longitudinal study of success versus failure in contraceptive planning. *J Population, 1*, 69-83.

Otto SJ, Houwelingen AC, Antal M, Manninen A, Godfrey K, Lopez-Jaramillo P, Hornstra A. (1997). Maternal and neonatal essential fatty acid status in phospholipids: an intenational comparative study. *Eur J Nutrd, 51*, 232-242.

Otto SJ, van Houwelingen AC, Hornstra G. (2000). The effect of supplementation with docosahexaenoic and arachidonic acid derived from single cell oils on plasma and erythrocyte fatty acids of pregnant women in the third trimester. *Prosatagland Leukot Essent Fatty Acids, 63*, 323-328.

Reese HW, Lipsitt L. (1970). *Experimental child psychology.* New York: Wiley.

Reynolds CR, Kamphaus RW. (2006). *Behavioral Assessment System for Children – 2.* Upper Saddle River, NJ: Pearson.

Ribisl KM, Walton MA, Mowbray CT, Luke DA, Davidson WS, Bootsmiller BJ. (1996). Minimizing participant attrition in panel studies through the use of effective retention and tracking strategies: Review and recommendations. *Eval Prog Plan, 19*, 1-25.

Richards JE, Casey BJ. (1992). Development of sustained visual attention in the human infant. In B. A. Campbell, H. A. Hayne, & R. Richardson (Eds.), *Attention and information processing in infants and adults: Perspectives from human and animal research* (pp. 30-60). Hillsdale, NJ: Erlbaum.

Richardson AJ, Puri BK. (2000). The potential role of fatty acids in attention-deficit/hyperactivity disorder. *Prostaglandins Leukot Essent Fatty Acids, 63*, 79–87.

Richardson AJ, Puri BK. (2002). A randomized double-blind, placebo-controlled study of the effects of supplementation with highly unsaturated fatty acids on ADHD-related symptoms in children with specific learning difficulties. *Prog Neuropsychopharmacol Biol Psychiatry, 26*, 233-239.

Richardson AJ. (2006). Omega-3 fatty acids in ADHD and related neurodevelopmental disorders. *Int Rev Psychiat, 18*, 155–172

Robbins TW, Everitt BJ. (1995). Arousal systems and attention. In M Gazzaniga (Ed.), *The cognitive neurosciences* (pp. 703-720). Cambridge: MIT Press

Roberts RJ, Pennington BF. (1996). An interactive framework for examining prefrontal cognitive processes. *Devel Neuropsychol, 12*, 105-126.

Ruff, H. A., & Lawson, K. R. (1990). Development of sustained, focused attention in young children during free play. *Devel Psychol, 26*, 85-93.

Salem N, Moriguchi T, Greiner RS, McBride K, Ahmad A, Catalan JN, Slotnick B. (2001). Alterations in brain function after loss of docosahexaenoate due to dietary restriction of n-3 fatty acids. *J Molec Neurosci, 16*, 299-307.

San Giovanni JP, Berkey CS, Dwyer JT, Colditz GA. (2000a). Dietary essential fatty acids, long chain polyunsaturated fatty acids, and visual resolution acuity in healthy full-term infants: A systematic review. *Early Hum Dev, 57*, 165-188.

San Giovanni JP, Parra-Cabrera S, Colditz GA, Berkey CS, Dwyer JT. (2000b). Meta-analysis of dietary essential fatty acids and long chain polyunsaturated fatty acids as the relate to visual resolution acuity in healthy preterm infants. *Pediatrics, 105*, 1292-1298.

Sawazaki S, Hamazaki T, Yazawa K, Kobayashi M. (1999). The effect of docosahexaenoic acid on plasma catecholamine concentrations and glucose tolerance during long-lasting psychological stress: a double-blind placebo-controlled study. *J Nutr Sci Vitaminol , 45*, 655-665.

Scott DT, Janowsky JS, Carroll RE, Taylor JA, Auestad, N, & Montalto MB (1998). Formula supplementation with long-chain polyunsaturated fatty acids: are there developmental benefits? *Pediatrics, 102*, E59.

Smuts CM, Borod E, Peeples JM, Carlson SE. (2003a). High-DHA eggs: feasibility as a means to enhance circulating DHA in mother and infant. *Lipids, 38,* 407-414.

Smuts CM, Huang M, Mundy D, Plasse T, Major S, Carlson SE (2003b). A randomized trial of docosahexaenoic acid supplementation during the third trimester of pregnancy. *Ob Gyn, 101*, 469-479.

Sorgi PJ, Hallowell EM, Hutchins HL, Sears B (2007). Effects of an open-label pilot study with high-dose EPA/DHA concentrates on plasma phospholipids and behavior in children with attention deficit hyperactivity disorder. *Nutr J, 13*, 6-16.

Spencer JP, Smith LB, Thelen E. (2001). Tests of a dynamic systems account of the A-not-B error: The influence of prior experience on the spatial memory abilities of two-year-olds. *Child Devel, 72*, 1327-1346.

Spreen O, Benton AL. (1963). *Sentence Repetition Test: Administration, scoring, and preliminary norms.* Unpublished manuscript, University of Iowa.

Stahl LA, Begg DP, Weisinger RS, Sinclair AJ. (2008). The role of omega-3 fatty acids in mood disorders. *Curr Opin Investig Drugs, 9*, 57-64.

Stevens LJ, Zentall SS, Abate ML, Kuczek T, & Burgess JR (1996). Omega-3 fatty acids in boys with behavior, learning, and health problems, *Physiol. Behav. 59*, 915–920.

Stordy BJ (2000). Dark adaptation, motor skills, docosahexaenoic acid, and dyslexia, *Am. J. Clin. Nutr. 71 (Suppl.)*, 323S–326S.

Stroop JR (1935). Studies of interference in serial verbal reactions. *J Exp Psychol, 18*, 643-662.

Thompson-Schill SL, Bedny M, Goldberg RF (2005). The frontal lobes and the regulation of mental activity. *Curr Opin Neurobio, 15*, 219-224.

Uauy R, Hoffman DR, Peirano P, Birch DG, Birch EE. (2001). Essential fatty acids in visual and brain development. *Lipids, 36*, 885-895.

Uauy RD, Birch DG, Birch EE, Tyson JE, & Hoffman DR (1990). Effect of dietary omega-3 fatty acids on retinal function of very-low-birth-weight neonates. *Pediatr. Res. 28*, 485–492.

Voigt RG, Llorente AM, Jensen CL, Fraley JK, Berretta MC, Heird WC. (2001). A randomized, double-blind, placebo-controlled trial of docosahexaenoic acid supplementation in children with attention-deficit/hyperactivity disorder. *J Pediatr, 139*, 189-196.

Wainwright PE, Huang YS, Coscina DV, Levesque S, & McCutcheon D. (1994). Brain and behavioral effects of dietary n-3 deficiency in mice: a three generational study, *Dev. Psychobiol. 27*, 467–487.

Waxman SR, Braun I. (2005). Consistent (but not variable) names as invitations to form object categories: New evidence from 12-month-old infants. *Cognition, 95*, B59 – B68.

Wenner JA, Bauer PJ (1999). Bringing order to the arbitrary: One- to two-year olds' recall of event sequences. *Inf Behav Devel*, *22*, 585-590.
Werkman SH, Carlson SE. (1996). A randomized trial of visual attention of preterm infants fed docosahexaenoic acid until 9 months. *Lipids, 31,* 91-97.
Weschler D. (2002). *Weschler Primary Preschool Scales of Intelligence – III.* Upper Saddle River, NJ: Pearson.
Willatts P (1999). Development of means-end behavior in young infants: Pulling a support to retrieve a distant object. *Devel Psychol, 35*, 651-667.
Willatts P, & Forsyth JS. (2000). The role of long-chain polyunsaturated fatty acids in infant cognitive development. *Prostaglandins Leukot Essent Fatty Acids. 63,* 95-100.
Willatts P, Forsyth JS, DiModugno MK, Varma S, Colvin M. (1998a). Effect of long-chain polyunsaturated fatty acids in infant formula on problem solving at 10 months of age. *Lancet, 352*, 688-691.
Willatts P, Forsyth JS, DiModugno MK, Varma S, Colvin M. (1998b). Influence of long-chain polyunsaturated fatty acids on infant cognitive function. *Lipids, 33*, 973-980.
Willatts P, Forsyth S, Mires G, Ross P. (2003). Maternal DHA status during late pregnancy is related to measures of infant look duration and acuity at age 4 months. *Soc Res Child Devel* (Abstract).
Willatts, P. (2002). Long chain polyunsaturated fatty acids improve cognitive development. *J Fam Health Care, 12,* 1474-9114.
Williams MA, Zingheim RW, King IB, Zebelman AM. (1995). Omega-3 fatty acids in maternal erythrocytes and risk of pre-eclampsia. *Epidemiology, 6*, 232-7.
Winefield AH, Winefield HR, Tiggemann M. (1990). Sample attrition bias in a longitudinal study of young people. *Austral J Psychol, 42*, 75-85.
Young CL, Dombrowski M. (1989). Psychosocial influences on research subject recruitment, enrollment and retention. *Soc Work Health Care, 14*, 43-57.
Young G, Conquer J. (2005). Omega-3 fatty acids and neuropsychiatric disorders. *Reprod Nutr Dev, 45*, 1-28..
Zelazo PD. (2006). The Dimensional Change Card Sort (DCCS): a method of assessing executive function in children. *Nature Protocols, 1*, 297-301.

H. Consortium/Contractual

Not applicable

I. Consultants

Not applicable.
