# Supplementary material for: Effect of Prenatal Docosahexaenoic Acid Supplementation on Blood Pressure in Children With Overweight Condition or Obesity: A Secondary Analysis of a Randomized Clinical Trial
Source: JAMA Netw Open. 2019 Feb 22;2(2):e190088. doi: 10.1001/jamanetworkopen.2019.0088 (PMC6484605; doi:10.1001/jamanetworkopen.2019.0088)
Supplement: Supplement 2. — eTable. Number of Completed Visits and Number and Percent of Usable Blood Pressure Assessments by Group and Age [file jamanetwopen-2-e190088-s002.pdf]

Supplementary Online Content

Kerling EH, Hilton JM, Thodosoff JM, Wick J, Colombo J, Carlson SE. Effect of prenatal docosahexaenoic acid supplementation on blood pressure in children with overweight condition or obesity: a secondary analysis of a randomized clinical trial. *JAMA Netw Open.* 2019;2(2):e190088. doi:10.1001/jamanetworkopen.2019.0088

eTable. Number of Completed Visits and Number and Percent of Usable Blood Pressure Assessments by Group and Age

This supplementary material has been provided by the authors to give readers additional information about their work.

©2019 Kerling EH et al. *JAMA Network Open.*

eTable. Number of Completed Visits and Number and Percent of Usable Blood Pressure Assessments by Group and Age

	Completed Visits		Usable Blood Pressure Assessment	
Age	Placebo	DHA	Placebo	DHA
	No.	No.	No.(%)	No.(%)
4 years	87	92	70 (80)	82 (89)
4.5 years	86	91	80 (93)	83 (91)
5 years	83	88	76 (92)	81 (92)
5.5 years	81	86	77 (95)	83 (97)
6.0 years	81	75	76 (94)	82 (98)

©2019 Kerling EH et al. *JAMA Network Open.*
