# Supplementary material for: Effect of Prenatal Docosahexaenoic Acid Supplementation on Blood Pressure in Children With Overweight Condition or Obesity: A Secondary Analysis of a Randomized Clinical Trial
Source: JAMA Netw Open. 2019 Feb 22;2(2):e190088. doi: 10.1001/jamanetworkopen.2019.0088 (PMC6484605; doi:10.1001/jamanetworkopen.2019.0088)
Supplement: Supplement 3. — Data Sharing Statement [file jamanetwopen-2-e190088-s003.pdf]

Data Sharing Statement

Kerling. Effect of Prenatal Docosahexaenoic Acid Supplementation on Blood Pressure in Children With Overweight Condition or Obesity. *JAMA Netw Open*. Published February 22, 2019. 10.1001/jamanetworkopen.2019.0088

Data
Data available: Yes
Data types: Deidentified participant data
How to access data: scarlson@kumc.edu
When available: With publication

Supporting Documents
Document types: None

Additional Information
Who can access the data: Researchers whose proposed use of the data have been approved
Types of analyses: Any research purpose or for meta-analyses
Mechanisms of data availability: without support but after approval
Any additional restrictions: None
